# Building Biological Relevance Into Integrative Modelling of Macromolecular Assemblies

**DOI:** 10.3389/fmolb.2022.826136

**Published:** 2022-04-11

**Authors:** Anne-Elisabeth Molza, Yvonne Westermaier, Magali Moutte, Pierre Ducrot, Claudia Danilowicz, Veronica Godoy-Carter, Mara Prentiss, Charles H. Robert, Marc Baaden, Chantal Prévost

**Affiliations:** ^1^ CNRS, Université Paris-Cité, UPR 9080, Laboratoire de Biochimie Théorique, Paris, France; ^2^ Institut de Biologie Physico-Chimique-Fondation Edmond de Rothschild, PSL Research University, Paris, France; ^3^ Biophysics and Modelling Department/In Vitro Pharmacology Unit–IDRS (Servier Research Institute), Croissy-sur-Seine, France; ^4^ Servier Monde, Suresnes, France; ^5^ Department of Physics, Harvard University, Cambridge, MA, United States; ^6^ Department of Biology, Northeastern University, Boston, MA, United States

**Keywords:** integrative modelling, biological function, large macromolecular assemblies, molecular dynamics simulations, normal modes, ryanodine receptor, homologous recombination

## Abstract

Recent advances in structural biophysics and integrative modelling methods now allow us to decipher the structures of large macromolecular assemblies. Understanding the dynamics and mechanisms involved in their biological function requires rigorous integration of all available data. We have developed a complete modelling pipeline that includes analyses to extract biologically significant information by consistently combining automated and interactive human-guided steps. We illustrate this idea with two examples. First, we describe the ryanodine receptor, an ion channel that controls ion flux across the cell membrane through transitions between open and closed states. The conformational changes associated with the transitions are small compared to the considerable system size of the receptor; it is challenging to consistently track these states with the available cryo-EM structures. The second example involves homologous recombination, in which long filaments of a recombinase protein and DNA catalyse the exchange of homologous DNA strands to reliably repair DNA double-strand breaks. The nucleoprotein filament reaction intermediates in this process are short-lived and heterogeneous, making their structures particularly elusive. The pipeline we describe, which incorporates experimental and theoretical knowledge combined with state-of-the-art interactive and immersive modelling tools, can help overcome these challenges. In both examples, we point to new insights into biological processes that arise from such interdisciplinary approaches.

## 1 Introduction

Many biological processes depend on the formation of large transient or permanent macromolecular assemblies. These assemblies may harbor signaling functions (e.g., membrane receptor proteins), act as motors for cell motility, membrane crossing, DNA maintenance, or ATP synthesis, or form scaffolds for the cell wall or for communication networks. In recent years, extraordinary advances have been made in both technology and information processing, leading to an avalanche of experimental 3D structures of large biomolecular complexes (Nogales, 2016; Murata and Wolf, 2018; Ziegler et al., 2021), many with resolutions better than 4 Å (Ghanim et al., 2021). The availability of these structures has enabled unprecedented advances in understanding many important biological processes (Verkhivker et al., 2021). It seems that access to high-resolution structures of all large multimolecular edifices involved in complex biological processes is only a matter of time. Does this mean that we will be able to understand their mechanisms? This question highlights a fundamental challenge for understanding functional biological assemblies: how to consistently integrate experimental structure determination into mechanistic and functional models. From this point of view, integrative modelling can be seen as a tool that goes beyond the structural level to explicitly include questions of biological function and mechanism.

Mechanistic understanding of the function of proteins and other biological macromolecules is usually based on the availability of three-dimensional structural information. The earliest example of this is probably the replication of DNA based on an atomic model of the double helix (Watson and Crick, 1953). Shortly thereafter, the first crystal structure of a protein, myoglobin, appeared (Kendrew et al., 1958), sparking a movement to determine more and more macromolecular structures at ever higher resolution by X-ray diffraction, neutron diffraction, NMR, and cryo-electron microscopy. Currently, there are nearly 200,000 experimental 3D structures of individual proteins and small complexes in the Protein Data Bank. Analysis of this rich source of high-resolution structural data increasingly contributed to the creation of models of proteins for which no experimental structure existed (e.g., Webb and Sali, 2021). This culminates today in the successful application of artificial intelligence to the automatic generation of highly accurate 3D models (Jumper et al., 2021), reflecting the tremendous progress that has been made in the last decade in predicting protein structures from sequences. Taken together, this detailed structural information has helped to elucidate the mechanisms of myriad catalytic reactions and biochemical processes carried out by macromolecules in the cell.

Over the same period, and largely thanks to advances in cryoEM (Nogales, 2016; Murata and Wolf, 2018) and its coupling with integrative modelling (Ziegler et al., 2021), this inexorable push towards high-resolution 3D structural data has attained the scale of large-scale assemblies in the cell. It would thus appear that understanding the mechanisms of these processes is indeed at hand. Yet deciphering the function of large assemblies in many cases defies the “structure illuminates function” logic. This may occur because 3D data is missing for particular regions of otherwise well-resolved structures, thus preventing one from fully exploiting them, or from the absence of essential cofactors or partner proteins needed for their function. Similarly, structural disorder in the complex may be intrinsic to its function, so that a structural average or snapshot provides little insight. The availability of the structures of the endpoints of a given process (reactants, products) and of a few intermediates may not be sufficient *per se* to directly address the underlying biological mechanisms. This could be because the stable intermediates are structurally too far apart to simply infer the transition between them, or because new structural information reveals new gaps in our knowledge. Structural sets could reflect different experimental conditions or different levels of resolution and thus not readily provide a meaningful overview of the biology in question. In such cases, filling the structural gaps requires the additional effort of integrating complementary experimental and numerical approaches.

The term *integrative modelling* is frequently used to describe obtaining 3D structures of macromolecular assemblies based upon medium-to low-resolution data such as cryo-EM maps or SAXS profiles. Additional information obtained from mutagenesis, NMR, or mass-spectrometry and cross-linking (Faini et al., 2016) has frequently been used to reduce structural ambiguities. Docking has been helpful in predicting 3D structures of large macromolecular complexes starting from those of their known constituents (Inbar et al., 2005; Schneidman-Duhovny and Wolfson, 2020), although many such cases remain a challenge due to complex combinatorial problems. Platforms for integrating such diverse information into structural models propose user-friendly software pipelines that take advantage of the known high-resolution 3D-structures of complex components or their homologs sharing a sufficient degree of sequence similarity (Russel et al., 2012; van Zundert et al., 2016; Mirabello and Wallner, 2017). The outcome of such modelling is often considered to represent metastable conformational substates that exhibit robust stability when subjected to hundreds of nanoseconds of molecular dynamics simulations. However, these criteria are not always sufficient to approach functional questions.

Here, we focus on two representative examples of assemblies where structural information alone is not sufficient to obtain mechanistic insights. The first is the ryanodine receptor (RyR). To date, RyRs are the largest known ion channels, with a molecular mass of over 2.3 MDa. Each subunit of the homotetrameric channel contains more than 5,000 residues. The number of RyR cryo-EM datasets is steadily increasing (Baldwin et al., 2018; Spurgeon et al., 2021). However, although many maps are available that span different conformational substates obtained under different conditions, there are still no complete models of this receptor. The maps lack homogeneity and the necessary coherence that would permit gaining insight into the structure-dynamics-function relationships in this critical channel protein and help uncover missing biological significance. The second case concerns the nucleoprotein filament complex that is active in homologous recombination (HR)– a fundamental biological process that aims at faithfully repairing broken DNA strands (Prentiss et al., 2015; Bell and Kowalczykowski, 2016). This complex comprises a long filament of recombination proteins assembled on DNA strands. The whole assembly undergoes very rapid dynamic evolution that is directly linked to its function, hence the difficulty in obtaining reliable structural information on intermediate states.

We illustrate how these challenges can be addressed using a modelling pipeline (Table 1) which begins with relatively simple structural modelling and gradually incorporates steps that require more sophisticated methodologies. The process is explicitly oriented towards arriving at dynamic information which may be used simply to verify the metastability of a state, for example, or to explore changes of the system in response to a perturbation or relaxation process. A given step in Table 1 may or may not be applied depending on the biological context. In the first system presented, the ryanodine receptor RyR1, we focus on the modelling of “building blocks” consisting of a single macromolecule but in different conformational states, for which coherent modelling, validation and comparisons can be performed. The second example, a RecA-DNA-polymerase complex that executes the final steps of the HR process, concerns the assembly and coordination of building blocks that are already available into functional biological machines that can be used to predict mechanistic aspects of the process. Although this is conceptually more complex than the construction of the building blocks themselves, the two examples share similar logic at different levels, and employ dynamic exploration as part of the modelling and preliminary exploration steps.

**TABLE 1:** Integrative modelling tasks applied to two representative examples.

	RyR1	RecA*
generation of missing atom coordinates	yes	no
side chain reconstruction	yes	no
backbone modelling	yes	yes
*ab initio* structure prediction	yes	no
interactive loop modelling	yes	yes
interactive engineering	no	yes
cofactor/molecular docking	no	yes
MD refinement	yes	yes
MD (meta-)stability verification	yes	no
MD/NM preliminary (or model) exploration	yes	yes

## 2 Methods

The elements described here allowed performing the integrative modelling tasks in our modelling pipeline, which are summarized in Table 1. We also detail the protocols we developed, introduce nomenclature, and explain key decisions. The overall methodology is general, but the actual details are specific to each system.

### 2.1 Full-Length RyR1 Models

#### 2.1.1 Multiple Alignments

Three mammalian isoforms of RyR exist in cells: RyR1 predominates in skeletal muscle; RyR2 is the most abundantly expressed isoform in cardiac muscle; and RyR3 is expressed differentially in brain (Takeshima et al., 1989; Nakai et al., 1990; Fill and Copello, 2002; Lanner et al., 2010), endocrine cells and other tissues. At least two of these isoforms, RyR1 and RyR2, share the same structural organization with an overall mushroom-like shape as observed by electron microscopy (Van Petegem, 2015; Zalk et al., 2015; Georges et al., 2016; Peng et al., 2016; Dhindwal et al., 2017). These isoforms have a high percentage (ca. 70%) of sequence identity.

We selected reviewed RyR sequences from the UniProtKB database (Boutet et al., 2016). The entries used for this study were: P11716, P21817, E9PZQ0, P30957, Q92736, E9Q401, Q9TS33, Q15413, and A2AGL3. ClustalO (Sievers et al., 2011) was used for alignments, WebLogo (Crooks et al., 2004) and Skyalign (Wheeler et al., 2014) were used to assess the presence of conserved residues.

#### 2.1.2 Secondary Structure and Transmembrane Domain Predictions

RyR1 homologues have a high percentage of sequence identity (on the order of 70%), and their secondary structures are well conserved. We were particularly interested in the interspersed disordered region between residues 4254 and 4539, which separates the cytoplasmic from the transmembrane domains. To obtain information on this region, which we call “Big Loop” (BL), we first examined its sequence conservation and residue composition. Since we focus on the RyR1 isoform, we used five homologous protein sequences and performed a multiple sequence alignment using the Clustal Omega software. Secondary structures were predicted using several web servers, such as PSIPRED and PSSPRED (Jones, 1999; Yan et al., 2013). Transmembrane regions were predicted using the TOPCONS and CCTOP servers (Dobson et al., 2015; Tsirigos et al., 2015). The ion binding sites were predicted via the IonCom program (Hu et al., 2016). For the BL, we used PEP-Fold3 (Lamiable et al., 2016), particularly for residues 4320 to 4345. Finally, we assessed the presence of an auxiliary transmembrane helix (or helices) and other secondary structure elements by visual inspection of the cryo-EM density maps.

#### 2.1.3 Starting Structures Used for Modelling

The partial structural models deposited in the PDB (Georges et al., 2016) were used as initial data for a series of modelling steps (Table 2). Since then, additional structures for the rabbit RyR1 isoform were released, which we may use in subsequent work to compare to the models that we produced. We briefly examined this new structural data, which only became available after our work was completed, and concluded that there was no imperative to repeat the procedure for the purpose of illustrating the integrative modelling pipeline.

**TABLE 2:** RyR1 putative functional states, conformations, ligands, PDB and EMDB identifiers used for this work. # AAs stands for number of amino acids in the corresponding sequence. CFF stands for caffeine. All data are for rabbit RyR1.

State	Conformation	Ligands	PDB Id	EMDB Id	# AAs
apo	closed	-	5TB0	8391	18096
primed	closed-like	Ca2+	5T15	8342	19136
Activated/intermediate	intermediate	ATP/CFF	5TAP	8381	18096
activated	open	ATP/CFF/Ca2+	5TA3	8377	18096
locked	open	Ryanodine	5TAW	8387	18096

#### 2.1.4 Initial Structure Preparation

Due to the inherent conformational flexibility of some regions of RyR1, the cryo-EM structures contain several gaps and are subject to many uncertainties regarding the coordinates of specific residues.

We first examined the coordinates of the “unknown residue” (*UNK*) annotations in the PDB files. We assumed that the C*α* coordinates in the PDB files were acceptable starting positions for these residues. These amino acids were assigned initial positions with ROSETTA based on their sequence and then checked visually one by one against the density map. Further, missing side chains and residues of known sequence were modelled with the *de novo* modelling method using the program ROSETTA remodel (Huang et al., 2011).

At the end of the model building process, we checked newly rebuilt residues visually, especially the helical domains such as the junctional solenoid (J-solenoid), using selected buried residues as a guide to assess whether the side chains of the residues fit well into the density maps.

Before refining the entire model, several loops or side chains were optimized by a combination of side-chain reorientation and loop placement using in-house routines for flexible and interactive molecular dynamics implemented in the BioSpring tool (Molza et al., 2014) followed by energy-minimization steps using the YASARA Structure software (version 17.12.24) (Krieger et al., 2002). This procedure was particularly useful for refining interfacial loops between adjacent subunits (residues 4290–4299, 3086–3120, 3067–3075, and 4346–4426).

#### 2.1.5 Model Refinement

For refining the coordinates of the modelled residues and verifying the side-chain placements, Molecular Dynamics Flexible Fitting (MDFF) simulations were performed using the NAMD software (Phillips et al., 2005; Trabuco et al., 2008). Our initial models were refined using the real-space refinement procedure from the PHENIX package (Afonine et al., 2013; Urzhumtsev et al., 2016) in order to avoid the propagation of geometry errors from the input structure (PDB and *de novo* modelling output files) during the MDFF procedure.

The secondary structures of residues folded into *α*-helices or *β*-sheets were preserved during simulations by imposing harmonic restraints with force constants of 20 kcal mol^−1^ rad^−2^ for dihedral angles and hydrogen bonds, involving backbone atoms of the same subset of residues using the ssrestraints command.

The NAMD configuration files for the MDFF simulations were generated using the mdff setup command. The MDFF simulation was performed *in vacuo* with NAMD 2.12 using the CHARMM36 force field (Lee et al., 2014). 10,000 minimization steps were performed, followed by slow heating to 300 K and 4 M production steps (4 ns) of molecular dynamics, with a time step of 1 fs/step. A scaling factor of 1 kcal mol^−1^ was used to adjust the strength of the influence of the electron density map on the tetramer model during the fitting process. The remaining parameters were defined based on default values in the mdff_template.namd file. The MD calculations were performed on the ADA supercomputer at the French IDRIS Supercomputing Center.

For reducing remaining outliers, additional refinement steps were performed using PHENIX and YASARA minimization steps under restraints.

#### 2.1.6 Side-Chain Refinement in Ligand Binding Sites

As starting positions, we used the coordinates of the calcium and zinc ions, ATP and caffeine molecules, as previously determined by electron microscopy (Georges et al., 2016). For ryanodine, we used a Ryd model provided by Ngo and the coordinates from their MD study of ligand placement (Ngo et al., 2017). The models were created using YASARA. For each model, the most likely protonation state was calculated at pH 7.0. The program YASARA was used for atom typing and hydrogen atom assignment, followed by virtual titration. Sidechains of residues forming ligand binding sites and ligands were then refined using a YASARA macro within the VINALS method (VINA with Local Search) for local ligand docking plus minimization steps. The simulation cell was enlarged by 10 Åin this step.

#### 2.1.7 Coarse-Grained Molecular Dynamics Simulations

Coarse-grained Molecular Dynamics (CGMD) simulations were performed using the Martini force field (http://www.cgmartini.nl/). The setup uses version v2.2 of the force field (de Jong et al., 2013) in combination with the ELNeDin elastic network (Periole et al., 2009) and the GROMACS 2016.3 simulation engine (Abraham et al., 2015). Proteins were embedded in a membrane composed of DOPC/DOPE at a ratio of 5:3, and the systems were neutralized and then solvated in 150 mM NaCl. More than 100K pseudo atoms were simulated for two systems containing the channel core (with or without the BL region). More than 438K pseudo atoms were simulated for the entire protein. The calculations were performed at the French GENCI “IDRIS” Supercomputing Center and on local GPU clusters.

#### 2.1.8 Normal Mode Analyses

Due to the large size of the RyR1 system, we carried out Normal Modes Analysis (NMA) using the Non-Linear Rigid Block (NOLB by Hoffmann and Grudinin (2017)) software, which uses a coarse-grained block approach (Durand et al., 1994). In addition to efficiently analyzing large assemblies, NOLB decomposes the block motions into instantaneous rotational and translational components, whose nonlinear extrapolation allows the computation of periodic trajectories for visualization that preserve structural integrity better than standard linear approaches. The 100 lowest frequency vibrational modes were calculated for each of the superimposed RyR1 conformations using an all-atom elastic network model with an interaction cutoff distance of 10 Å. Ligand atoms, when present, were included in the analyses. Vibrational modes were sorted in order of frequency $\sqrt{\lambda_{i}}$ , with *λ*_*i*_ being the eigenvalues of the mass-weighted stiffness matrix (Hoffmann and Grudinin, 2017).

Two NM sets *a* and *b* were compared by examining the subspace overlap, or sum of squared projection of each mode vector from set *a* onto the space spanned by the modes of set *b* (see, e.g., Batista et al., 2010)]. Only *C*_*α*_ atom movements were used for these comparisons. The comparison subspace was obtained by orthonormalizing the *C*_*α*_ mode vectors from set *b* using standard techniques. The value of each vector overlap lies between 0 (no subspace overlap) and 1 (perfect overlap).

NOLB was also used to compute transition paths between pairs of RyR1 conformations by minimizing the RMSD between two input structures (an initial and a target structure) obtained by the linear combination of the calculated modes; these paths were achieved using all 100 computed modes. Scripts were written in Python (general numerical analysis and Chimera scripts) and Tcl (in the VMD environment) to analyze the NM results.

### 2.2 Atomic Model of a RecA*-DinB Complex

#### 2.2.1 Starting Point for the Present Study

**FIGURE 1:**
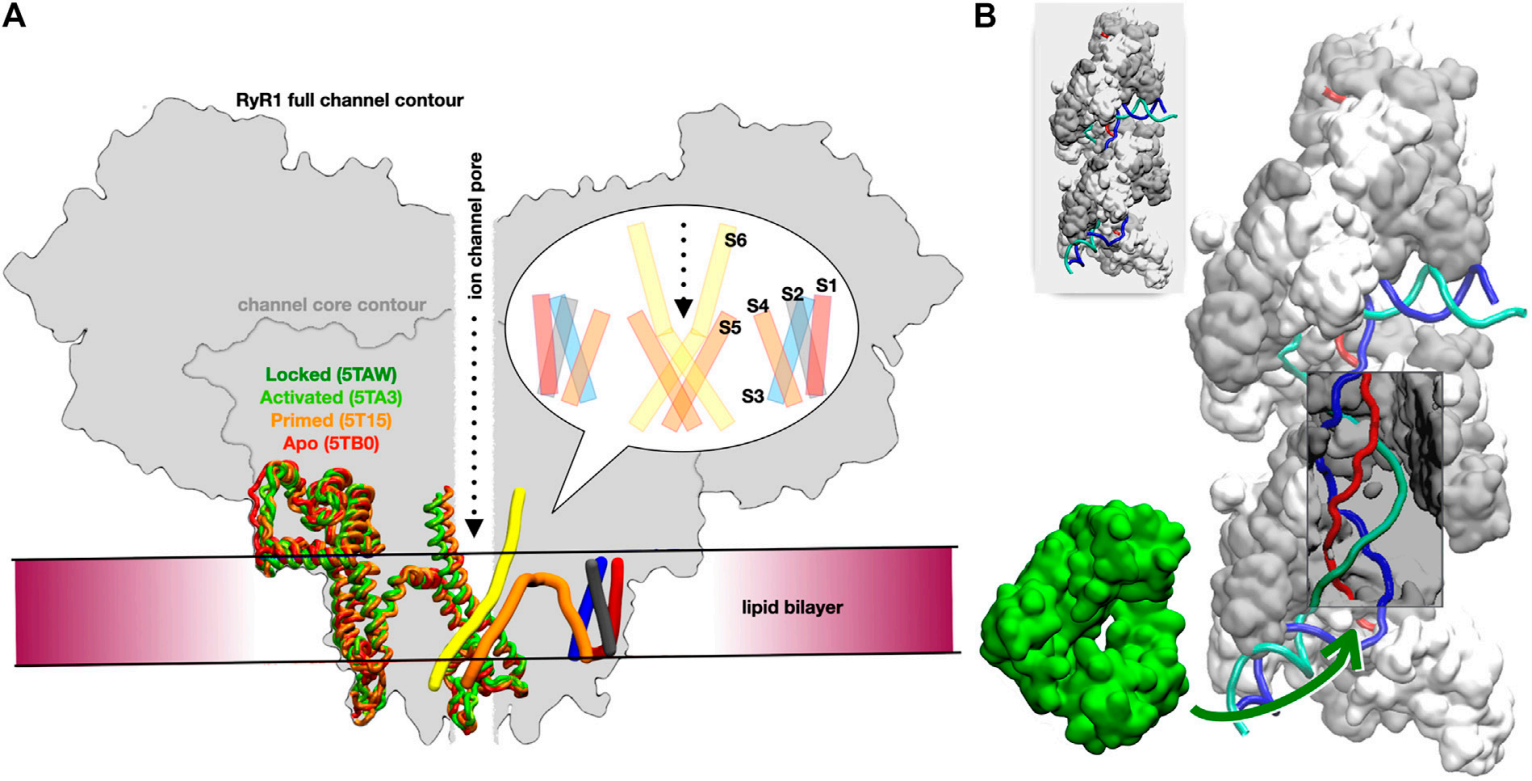
Overview of the two studied systems. **(A)** Schematic cross-sectional view of the full RyR channel model. The approximate location of the lipid bilayer is drawn, as is the central ion channel pore. On the left side of the schematic, the helical sections for a transmembrane domain monomer for each of the four states locked (dark green), activated (light green), primed (orange), and apo (red) are overlaid to highlight the conformational changes in this region. On the right side, the pattern of the six transmembrane helices is shown in cylindrical form: S1 in red, S2 in grey, S3 in blue, S4 and S5 in orange, and S6 in yellow. A bubble inset shows a schematic cross-section of the transmembrane domain, as typically found in the literature, for reference. **(B)** Two turns of the RecA* filament with three bound DNA strands; RecA proteins are represented alternatively in white and grey (surface); the DNA strands are schematically represented in red, blue and cyan; the section that is incorporated in the filament form a D-loop, where the blue strand has exchanged its pairing partner from the cyan to the red strand; a window has been opened in the filament to visualize the otherwise hidden D-loop (a complete filament is represented in the top left insert). The DinB DNA polymerase is represented in green surface; the green arrow points to the region of the filament where DinB binds to the DNA strands and RecA monomers in order to start elongating the red DNA strand.

The starting point for this system was the results of a preliminary coarse-grained study of the densely packed association complex between the RecA nucleoprotein filament (RecA*) and DNA-polymerase IV (DinB), which had been obtained by interactive simulations using the BioSpring software also used for the RyR1 modelling above (Section 2.1.4), and which represents each component of a complex system by a variable network of harmonic springs, allowing interactive dynamic testing of possible component orientations and deformations (Molza et al., 2014; Tashjian et al., 2019). In this case the components were the nucleoprotein filament (RecA and DNA) resulting from our earlier strand exchange simulations (Yang et al., 2015) and the crystal structure of DinB bound to its cognate DNA (PDB code 4IRC) (Figure 1B).

#### 2.2.2 Initial Model Refinement

The result of the coarse-grained BioSpring flexible assembly process required refinement, as much of the DinB secondary structure was distorted due to the spring network adaptation to the crowded environment of the RecA* filament. We thus superimposed individual DinB secondary structures taken from the crystal structure of DNA-bound DinB onto the corresponding regions in the preliminary model. One helix showed a partial steric clash with the terminal RecA monomer; the clash was released by laterally displacing the helix as a rigid body by 2 Å with respect to the corresponding BioSpring-positioned segment. Where possible, the loops linking secondary structure regions in the crystal structure were included in the model when this resulted in no steric clashes with RecA proteins; otherwise the BioSpring loop structure was used. All disruptions of the peptide chain structure after this process were removed using energy minimization with NAMD, which restored the standard covalent bond geometry.

#### 2.2.3 Introduction of a Guanine Nucleotide Tag

We introduced a guanine nucleotide (resid 11 in the template strand, noted $G_{11}^{\text{templ}}$ ) as a tag in the template strand in order to be able to identify the site of nucleotide (CTP) addition. The final simulated complex was formed by 17 RecA monomers, the DinB protein and three DNA strands forming a D-loop inside the RecA filament: the ssDNA or primer, with sequence (5′–3′) (dT)_53_, the template strand (dA)_10_dG(dA)_63_ partly dissociated from the homologous dsDNA, and the displaced strand (dT)_74_ from the dsDNA.

#### 2.2.4 All-Atom Molecular Dynamics Simulation

MD simulations were performed with NAMD 2.10 (Phillips et al., 2005) and the CHARMM 27 force field with CMAP corrections (Mackerell et al., 2004). A 2 fs integration timestep was used with the SHAKE algorithm, long-range electrostatics were accounted for using the particle-mesh Ewald method, and a Nosé-Hoover-Langevin piston was used for pressure maintenance. The complex was solvated in a TIP3P water box with 0.15 mMol NaCl and progressively heated to 300K. During heating, C_*α*_ and P atoms were harmonically restrained to their initial position with a force constant of 0.5 kcal mol^−1^ Å^−2^ that was gradually released during 20 ns equilibration followed by a 270 ns production phase. Calculations were performed at the French GENCI CINES Supercomputing Center.

## 3 Results

In this section we describe how we created models using our integrative modelling pipeline by detailing the modelling tasks summarized in Table 1 employed for each of our two example systems. We then present an initial set of interpretations of the models’ properties to show how they can provide biologically useful new information. The first “use case,” RyR, illustrates how consistent integrative models of a single macromolecular building block can be created. The second use case, RecA, documents how to create larger assemblies from such building blocks and the pitfalls to avoid to create a functionally relevant model. The overall approach, however, is general. The details provided for each system are intended to support the usefulness of the models and to provide avenues for further exploration of these biological systems. These may be reported in future work.

### 3.1 Modelling the RyR1 Ryanodine Receptor Channel and Its Dynamics

Ryanodine Receptors (RyRs) regulate cytosolic calcium concentration, which is critical in numerous signaling pathways. Mutations in these receptors in muscle cells can lead to severe skeletal muscle and heart disease. RyRs belong to the six-transmembrane helix ion channel superfamily (Lanner et al., 2010) and are localized in the endoplasmic and sarcoplasmic reticulum.

Here, RyR1 provides a case study to demonstrate general aspects of the pipeline that lead from deposited cryo-EM datasets to complete models that can be used for molecular modelling studies on mechanistic and structural aspects. We were particularly interested in 1) addressing issues related to the channel’s large size and 2) benefitting from the availability of maps for multiple functional/conformational sub-states, which provide a good test for the usability of the final models and the level of detail and accuracy that can be expected from them.

We focused on the rabbit RyR1 isoform due to the availability of numerous structural and experimental studies on its gating and activation (Georges et al., 2016). Our goal was to test whether reliable atomistic models of the five resolved channel states (Table 2) could be constructed and mechanistically interpreted based on the experimental data. These five states are defined according to Georges et al. (2016): for the apo and primed states, the channel is in a closed conformation, whereas in the activated and ryanodine-locked states, it is in an open conformation. Figure 1A provides a schematic view of the different states of the receptor highlighting the transmembrane and adjacent regions (the intermediate state is not shown in order to reduce visual distraction).

#### 3.1.1 Multiple Sequence Alignment

As previous comparative studies have shown (Yan et al., 2015; Efremov et al., 2015), RyR homologs have a significant percentage of sequence similarity, especially in structured regions such as the solenoid domains, with the highest sequence identity in the transmembrane domain (TMD). For example, human and rabbit RyR1 sequences are found to have 96.6% identity. Clustal Omega analyses revealed a high degree of similarity for the RyR isoforms RyR1 to RyR3 with percentages of more than 73%, while the identities are slightly lower at about 66%. In the divergent regions observed in the multiple alignments, more than 100 residues in the RyR3 sequence between the SPRY2 and SPRY3 domains are absent. Interestingly, even within these divergent regions, some conserved amino acids were highlighted. For example, a glutamate-rich loop (residues 1875–1921), in which some charged residues are conserved (particularly in the sequences of the RyR1 isoform) (Supplementary Figure S1A) or the Big Loop (BL, residues 4254–4539) connecting the cytoplasmic and transmembrane domains (Supplementary Figure S2). DISOPRED (Ward et al., 2004) predicted the BL region to be unstructured. Moreover, it was not resolved in any cryo-EM electron density map available when constructing our models. These regions have a particular amino acid composition that we analyzed further. A sequence logo plot clearly shows pronounced conservation within the glutamate-rich region of the loop at residues 1875 to 1921. For the disordered BL loop (residues 4254–4539), the sequence logo shows a variety of features, for example, the presence of a putative PE_5_ Ca^2+^binding site motif, hydrophobic and charged residues with a polyarginine repeat (that are either conserved or share considerable similarity), and many glycine residues known for their flexibility (Supplementary Figure S1B). The multiple sequence alignment with Clustal Omega revealed a high percentage of residue conservation for the BL region in homologs: for example, a 94% sequence identity for RyR1_BL human and RyR1_BL rabbit.

#### 3.1.2 Clues for an Additional Transmembrane Helix in the Big Loop Region

The multiple sequence alignment raises the question of the structural interpretation of the BL residue range (4254–4539). We wondered about the role of this disordered region connecting cytoplasmic and transmembrane parts. Since no experimental structural data was available, we performed an *in silico* study of this region. To this end, we analyzed the intrinsic disorder and predicted possible secondary structure elements as well as the transmembrane region, which indicated the possibility of an additional transmembrane helix in the BL region (Supplementary Figure S3). When we began this study, ambiguous information about the presence of a transmembrane helix was available from cryo-EM. Below, we review the various observations from our investigations that support such a finding.

##### 3.1.2.1 Secondary Structures and Propensity to Embed into the Membrane

PPSIPRED predicted five putative alpha-helices within the big disordered region (see Supplementary Figures S3A,B). The TOPCONS and CCTOP server predictions for the transmembrane region predicted multiple transmembrane parts. Interestingly, most tools predicted the same auxiliary transmembrane helix in the region of residues 4320 to 4336 as shown in Supplementary Figure S3C. To evaluate these results, we attempted to model some parts of this loop using a *de novo* approach with the PEP-FOLD3 package (Lamiable et al., 2016).

##### 3.1.2.2 Structural Predictions Through *De Novo* Folding

The Pep-Fold3 predictions indicate that the conserved regions (residues 4310–4318 and other parts of the BL region previously shown in the sequence logos in Supplementary Figure S1B) are most likely in helical conformation (Supplementary Figure S3D).

##### 3.1.2.3 Unravelling the Role of the BL Region

The results and predictions for the transmembrane region are consistent with the literature, which suggests that some residues in this region tend to fold into an alpha-helix and possibly embed into the membrane (Van Petegem, 2015; Zalk et al., 2015; Du et al., 2002; Van Petegem, 2017; Santulli et al., 2018; Efremov et al., 2015) (Supplementary Figure S3C). An additional arginine-rich helix could be localized at the surface of the bilayer (Hristova and Wimley, 2011; Ulmschneider et al., 2017). The BL region could be interacting with other partner molecules or phospholipids or even be implicated in the oligomeric interactions of the RyR. The propensity of some residues to fold into transient TM helices could also impact function, but this aspect remains somewhat unclear. These hypotheses should be verified experimentally. Comparing the rabbit and human RyR sequences, we can speculate about the role of the BL region. Because of the high identity and similarity percentages, we could use the initial models for this region as structural templates for constructing full-length RyR2 and even RyR3 models.

#### 3.1.3 Model-Building Procedure

Despite many available maps for RyR, complete models of the protein are not yet available. This lack of completed models hampers many efforts to obtain insight into the structure-dynamics-function relationship. In addition, RyR serves here as a use case to show how information from the pool of deposited cryo-EM datasets can be used to complete these models. Given that the number of relatively high-resolution datasets is continuously increasing, we hope that the model building procedures developed here will provide a general reference protocol that can be further adapted and optimized for exploring mechanistic and structural aspects of other systems under investigation.

**FIGURE 2:**
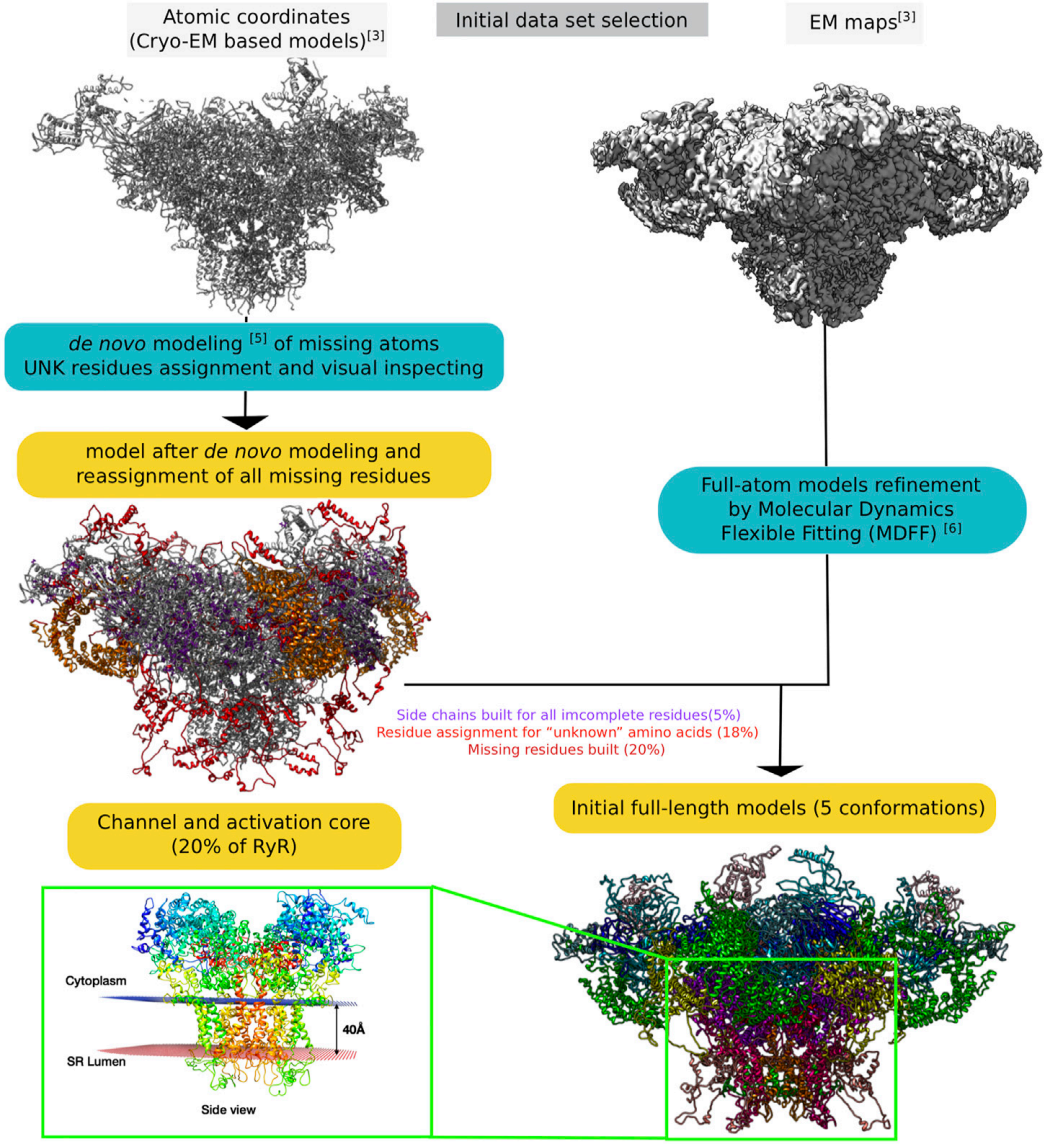
Schematic representation of the entire process of reconstructing the RyR1 model from raw electron microscopy maps to full-length whole-atom models.

Figure 2 provides a global schematic overview of the entire modelling procedure. In the following sections, the individual steps for the reconstruction of the receptors are described in detail.

##### 3.1.3.1 Full-Length Models of the Receptor (RyR1) at the Atomic Level

To model missing parts of the protein, several software packages were tested. For the specific use case of the RyR protein, given the challenges presented by its size, the best results were obtained by *de novo* modelling using Rosetta (https://www.rosettacommons.org/) to complete the models. The process is shown schematically in Supplementary Figure S4.

To optimize the geometry of these initial models for further computations after *de novo* modelling, we applied some model refinement routines using minimization steps within the Yasara software (Yasara Structure 17.12.24; http://www.yasara.org/) to remove possible clashes, knots, and some bond errors. We then refined the models for all atoms using the MDFF method (https://www.ks.uiuc.edu/Research/mdff/). This step is intended to optimize the newly created loops and side-chain coordinates based on the volumetric data from the experimental electron density maps.

**FIGURE 3:**
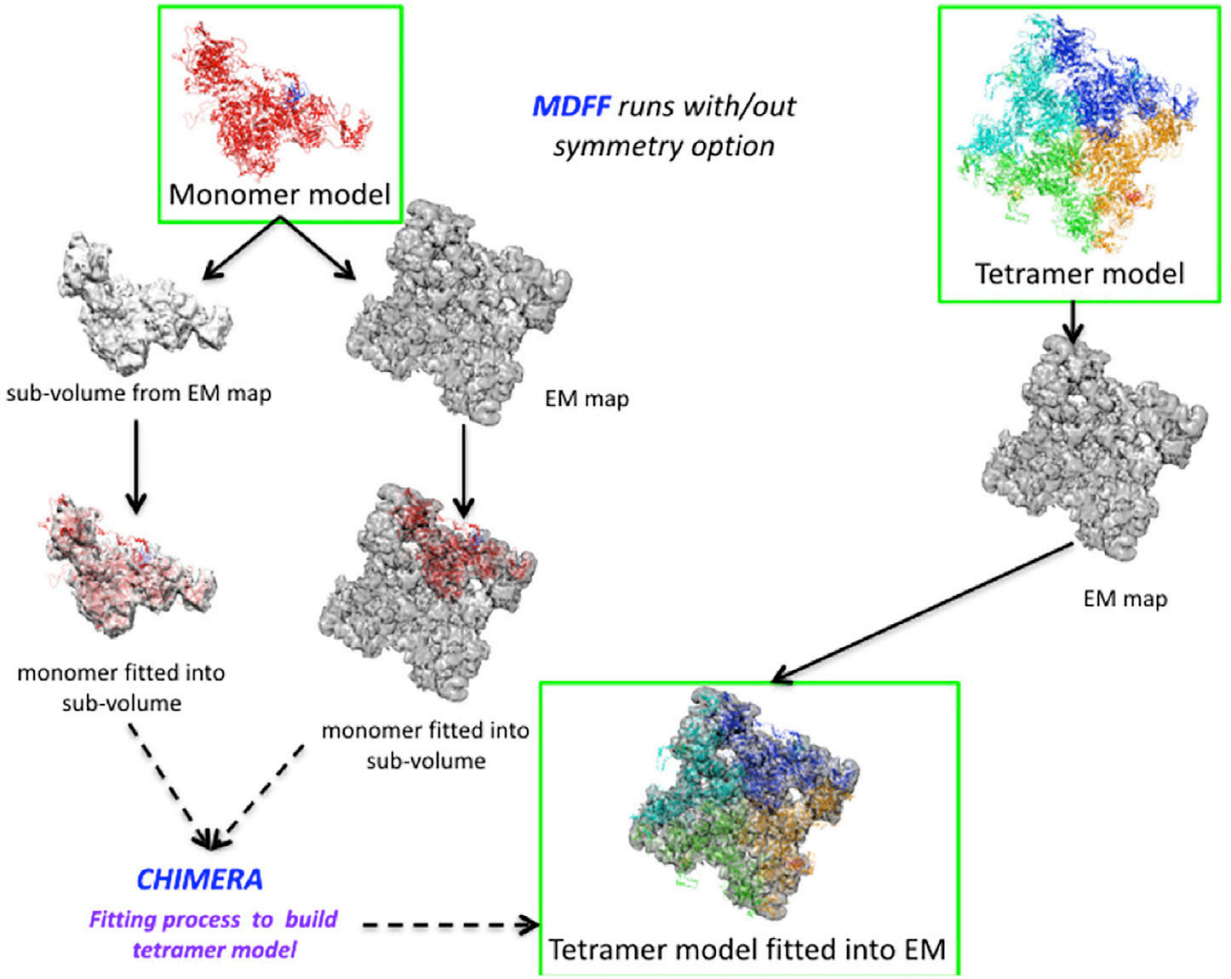
Runs performed to produce the MDFF calculations. Two different configurations are described in the Figure, either by fitting the monomer model to a partial volume or by fitting the tetramer model to the entire density map. The MDFF runs outlined with green rectangles represent a good compromise between the fit achieved and time to solution (left path) or a more direct, but computationally intensive way (right path) to obtain a tetramer model fitted to experimental maps.

We set up an MDFF routine that allows us to extract a partial volume corresponding to a monomer from a map to fit the model (Figure 3). After applying the MDFF procedure to a monomer, the different partial volumes fit well into the full map. However, we found that long loops (e.g., the region of residues 4254–4539) did not converge into the map despite many MDFF runs (Figure 3 and Supplementary Figure S5A). We attribute this lack of convergence to either the large size of these loop regions or to missing densities. Using the BioSpring tool (Molza et al., 2014) at a later stage (see below) improved convergence.

A variant of the MDFF method was required due to the system’s size and the computational resources needed. We experimented with a more computationally intensive protocol to fit the entire tetramer model into the map. Fitting each monomer into a subvolume is a good choice for a big system. However, if sufficient technical resources are available, fitting the whole system is preferable, as it also accounts for the interactions between the subunits.

The protocol is shown in Figure 3. It can be summarized for the monomer case as follows: Preparing the subvolume, fitting the monomer model to the subvolume and generating the tetramer model, checking that there is no overlap between subunits, and making any necessary corrections. For modelling the full tetramer (without the option of imposed symmetry), one first has to prepare the tetramer model and files for MDFF, then fit the model to the experimental map (in our experiments, this took about eight times longer than in the monomer case), check that there are no “aberrant” fits, and adjust the coordinates if necessary.

This step was followed by a visual inspection of the residues, especially residues modelled from “UNK” residue types. Some limitations are due to low-density regions or difficulties in fitting long loops. When necessary, some loops (e.g., in residue regions 4290–4299, 3064–3133) were shifted using the interactive and flexible method BioSpring previously developed in the laboratory (Molza et al., 2014), as shown in Supplementary Figure S5B. Namely, these regions were aligned to the corresponding densities of the neighbouring subunits.

In our final models used for subsequent analysis, the additional transmembrane helix was included for the apo, activated and locked states based on the evidence we found in the cryo-EM densities, whereas there was no density to model it into the primed and activated/intermediate states, despite the fact that the helix was present in PDB 5TAP.

##### 3.1.3.2 Assessment of Stereochemical Model Quality

Molprobity (Williams et al., 2018) was used to assess the structural quality of the models compared to the starting PDB structures, distinguishing the full models from those (“no-loops”) without modelled loops (red regions of Supplementary Figure S4). The results are summarized in Table 3 and show systematic improvement for some metrics, whereas others fluctuate or degrade. The C_*β*_ outliers in the 5tap conformation were corrected by our procedure, and rotamer outliers were systematically reduced for all models and fall within the recommended limit of 0.3. While the backbone outliers would normally be less than 0.2%, we observed several cases with higher values. The percentage in favoured Ramachandran regions should be above 98%, but does not go beyond 91.25% in the deposited structures in the PDB or in our models. In summary, despite the addition of a large number of missing structural data, the model quality did not significantly degrade, and even systematically improved for some metrics.

**TABLE 3:** Molprobity validation results (% residues) for the models derived from the five structural RyR1 templates used.

State	Structure	Favored regions	Allowed	Outliers	Rotamer outliers	C_*β*_ outliers
apo	5tb0	88.80	11.08	0.12	0.50	0
	noloops model	88.52	11.20	0.28	0.20	0
	full model	86.92	12.75	0.33	0.19	0
primed	5t15	88.24	11.49	0.27	0.45	0
	noloops model	91.25	8.51	0.24	0.06	0
	full model	89.42	10.33	0.25	0.05	0
activated/	5tap	89.92	9.90	0.18	0.54	4
intermediate	noloops model	89.21	10.46	0.33	0.26	0
	full model	87.18	12.47	0.35	0.22	0
activated	5ta3	89.09	10.76	0.15	0.54	0
	noloops model	89.40	10.36	0.24	0.17	0
	full model	87.32	12.41	0.27	0.17	0
locked	5taw	89.53	10.30	0.17	0.39	0
	noloops model	90.85	8.95	0.20	0.21	0
	full model	88.96	10.77	0.27	0.23	0

##### 3.1.3.3 Assessment of the Overall Assembly Through Coarse-Grained Simulations

The modelling steps described above resulted in all-atom structures of the RyR channel that were significantly enhanced with respect to the starting structures (Supplementary Figure S6A).

An important question is whether the molecular assemblies generated constitute a viable tetrameric channel in a membrane environment. To address this question, we performed Coarse-Grained Molecular Dynamics (CGMD) simulations. Two types of models of different sizes were used for this study: a **channel core model** centered on the RyR channel and a **full-length model** of the receptor. The channel core includes the core solenoid, the transmembrane (including the pore), and the C-terminal domains; the interaction of all these domains forms the ligand-binding sites of RyR1. This system comprises residues 3667 to 5037 (biological numbering) of each monomer. The complete model contains all residues in the RyR protein (residues 1 to 5037 for each monomer). To avoid bias in the coarse-grained simulations, we experimented with the presence or absence of the unstructured BL region that connects the cytoplasmic with the transmembrane domain. No connections or restraints were added between the monomers of the channel in either model.

Microsecond-scale CGMD simulations of the full RyR channel in DOPC/DOPE membrane and water simulations (500,000 particles, Supplementary Figure S6B) showed good stability over this period. A plot of the center-of-mass distance for all pairs of subunits is shown in Supplementary Figure S6C. Extended CGMD simulations (1–10 μs) were performed for the RyR1 channel core (3667–5037 of each monomer), the channel core excluding the BL region (deleted residues: 4254–4539) for the five RyR channel states of Table 2, and the full-length apo-state model. The protein architecture remained stable during the simulations, as shown in Supplementary Figure S6, confirming the interactions between the subunits of the tetramer and thus indirectly validating the model reconstruction and monomer interfaces. Specifically, the six helices (Figure 1) maintained their integrity during MD, with the highest fluctuation amplitude in the loop regions, especially in the S1-S2 loop. The distance between each monomer of the channel remained stable, with the radius of gyration varying between 10.6 and 10.8 nm (the variation may be due to the interaction with lipids). The bilayer thickness fluctuated little (38.7 ± 0.3 Å). In the future, we plan to transfer the systems from CG to the atomistic level and perform molecular dynamics for all atoms to achieve an accurate structural characterization of the RyR1 dynamics.

#### 3.1.4 Exploration of the Dynamics of the RyR Channel Models

##### 3.1.4.1 Studying RyR1 Deformations Through Normal Mode Analyses

Normal modes analysis (NMA) allows one to examine the dynamics of a given structure given the 3D coordinates of its component atoms, which in this case are obtained from our models. NMA is based on a harmonic approximation of the molecule’s potential energy in the region of a minimum in the potential energy surface, which is expressed as a function of changes in the 3*N* coordinates of the structure’s *N* atoms. It provides a detailed description of the vibrational dynamics associated with small perturbations of the minimum-energy structure. NMA notably provides a set of normal mode vectors, each of which consists of 3*N* components representing the amplitudes of atom movements for a single vibrational mode, plus the set of associated frequencies or deformation energies. In practice, the low-frequency (or low-energy) modes are of the most interest, as they have been extensively correlated with directions of conformational changes and dynamics in biological macromolecules (e.g., Tama and Sanejouand, 2001; Rueda et al., 2007).

Here we used NMA to better understand the dynamics of our models of the ryanodine receptor in its different conformational states and how these movements differ from one state to another. For such large molecules, the analyses were facilitated through the use of the NOLB software (Hoffmann and Grudinin, 2017). We performed NMA on the full-length RyR-1 models and the restricted channel regions after removing the modelled loop regions (structure “no-loops” in Table 3). These calculations were carried out on the five conformational states of RyR-1 (Table 2): the closed or closed-like states apo and primed, the intermediate state, and the open states activated and locked.

A variety of definitions for RyR’s motions have been used in the literature. Here we defined specific geometric characteristics of protein subdomain movements that characterized RyR vibrational modes. Three types of movements in particular were used to categorize the motions observed when visualizing the NM vibrational modes, and are designated here as twisting, breathing, and blooming motions. Supplementary Figure S7 shows schematic diagrams of these motions in RyR.

##### 3.1.4.2 Contributions of NM Movements to RyR Conformational Transitions

For the following analyses, we used the models restricted to the channel region, which encompasses residues 3667 to 5037 of each chain. The structural similarity of the different channel states was assessed by the *C*_*α*_ RMSD after superposition for all model structure pairs (Table 4). Of the channel regions of the five models, the primed and intermediate structures are the closest (1.4 Å*C*_*α*_ distance), followed by the models of the locked and activated states (1.6 Å), respectively. All other model structure pairs are between 2.4 and 2.9 Å apart.

**TABLE 4:** *C*_*α*_ rmsd in Angstroms for pairs of RyR model channel core models (calculated without the flexible BL region).

	Apo	Primed	Activated/intermediate	Activated	Locked
Apo	-	2.8	2.5	2.7	2.9
Primed		-	1.4	2.5	2.6
Activated/intermediate			-	2.4	2.5
Activated				-	1.6

**FIGURE 4:**
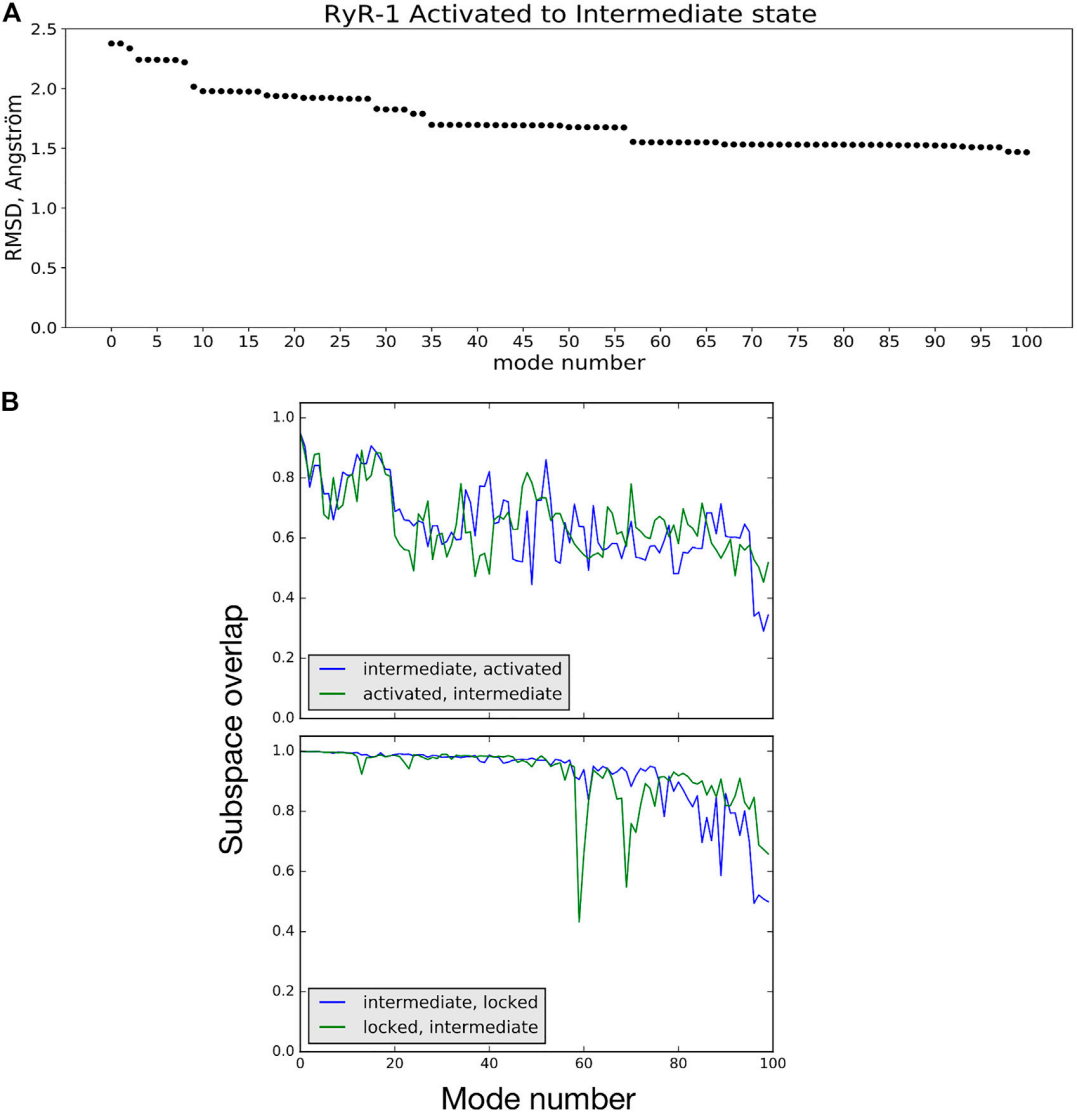
**(A)** RMSD between the activated and intermediate conformational states is reduced by 38% by displacing atoms along a linear combination of NM vectors. Mode 0 refers to the initial RMSD between the two model structures. **(B)** Subspace-overlap analysis for the 100 lowest-frequency normal mode vectors of selected pairs of conformational states of the RyR1 receptor. *Top:* In blue, overlap of each intermediate state mode on the subspace of the 100 activated-state modes. In green, overlap of each activated state mode on the subspace of the 100 intermediate-state modes. *Bottom:* Same coloring as in *Top*, but for the intermediate and locked state modes.

NMA suggests how the vibrational dynamics of the macromolecule may contribute to a given conformational transition. One way to see this is by calculating the linear combination of low-frequency vibrational mode vectors (atom movements) of the starting structure *A* that minimizes the least-squares distance to the final structure *B* (Hoffmann and Grudinin, 2017; Moal and Bates, 2010). Figure 4A shows an example of using this approach to studying the activated → intermediate transition The linear combination of normal-mode vectors for the 100 lowest-frequency modes of the activated conformation reduces the RMS distance to the intermediate conformation from 2.38 to 1.46 Å (38%), while the first 50 reduce the RMSD by 29%. As is often the case for conformational changes in biological macromolecules (Moal and Bates, 2010), a relatively small number of low-frequency NM movements participate in this transition; mode 9 alone contributes 10% of the total RMSD decrease. A short animation of the motion described by the low-frequency NM contributions to this transition is available (Robert and Molza, 2021). These NM contributions describe a longitudinal compression of the channel: bending of helix S6c and movement of the C-terminal domain act to reduce the opening on the cytoplasmic side, while the transmembrane domain twists about the channel axis and descends relative to the rest of the receptor, reducing the volume of the central cavity enclosed by helix S6 (breathing motion).

The NMA thus showed that the intrinsic, low-energy vibrational movements of the RyR channel in the active conformation already encode atom movements that tend to lead to a conformational transition to the intermediate state. This could be then used to obtain further insight into associated features such as domain movement, ligand-binding cavity opening/closing, etc. The NM motions thus reveal an energetic and dynamic rationale for previous depictions of conformational differences between cryo-EM structures (Georges et al., 2016; Willegems and Efremov, 2018), depictions that alone do not provide information for judging the energetic or dynamic favorability of inferred movements.

##### 3.1.4.3 Comparing Vibrational Motions of the RyR1 Channel as a Function of State

NMA can also be employed to assess the similarity of two conformational states of a protein in terms of their vibrational dynamics. To demonstrate this, we extracted the movements of common *C*_*α*_ atoms from the all-atom modes of different channel models calculated using NOLB. These will be referred to as *C*_*α*_ modes in the following. The plots shown in Figure 4B compare the intermediate and activated state models (top) and the intermediate and locked state models (bottom). In these plots, each of the 100 *C*_*α*_ mode vectors from one model *i*, arranged in order of increasing frequency, is projected onto the space spanned by the first 100 vibrational modes of the second model *j*, and vice versa. The intermediate and active state models can be seen to share about 70% of their low-frequency mode subspaces. On the other hand, the intermediate and locked states share a higher percentage of their low-frequency vibrational movements, particularly for the first 50 modes. This example demonstrates that comparing the vibrational dynamics of coherent models of the different states of the RyR receptor clearly provides a richer comparison than simple rmsd: the intermediate state is about the same rms distance from the activated state (2.4 Å) as it is from the locked state (2.5 Å), but the differences in vibrational dynamics of the models are more nuanced. Further investigation into comparisons of the RyR1 vibrational dynamics as a function of state are ongoing.

### 3.2 Integrative Modelling of DNA Polymerase Binding to RecA Nucleofilaments

We now provide an example in which refinement and initial validation of a structural model and dynamic exploration of the model go hand in hand. Here, the challenge is to validate the construction of an intermediate species in the final stage of homologous recombination (HR): the coupling of RecA-induced DNA strand exchange to DNA synthesis. As will be seen, the dynamic exploration is performed using classical molecular dynamics simulations rather than the harmonic dynamics (normal modes) approach employed above for the ryanodine receptor. Molecular dynamics appears to be well adapted to probing the modelled intermediate in this highly dynamic process because of the strong mechanical coupling among the components.

It is useful at this point to briefly present the HR process, largely conserved from prokaryotes to humans, along with the key players of this process relevant to the present modelling. Homologous recombination is a highly complex, multicomponent process that intervenes in the cell to assure the repair of double-strand DNA breaks, which are typically lethal (Bell and Kowalczykowski, 2016). The two broken ends are first processed to form a single-stranded DNA tail (ssDNA) on either side of the break. To repair the break, a helical nucleoprotein filament is formed by the oligomerization of protein monomers on these ssDNA tails. In bacterial HR, a well-studied model system, this protein is RecA. The nucleoprotein filament systematically binds and searches double-stranded DNA (dsDNA) in the genome to find a sequence match with the ssDNA. Within the HR filament, the two DNA species (ssDNA and dsDNA) are positioned near each other, allowing mutual probing of their sequences for homology, followed by base pairing and strand exchange in the case of homology (Prentiss et al., 2015; Bell and Kowalczykowski, 2016). This probing and proofreading process takes advantage of frequent reversals of strand exchange (Danilowicz et al., 2021), but this phase terminates when a DNA polymerase takes over, pushing the strand exchange reaction towards irreversibility (Lu et al., 2019) by using the dsDNA complementary strand as a template to start elongating the ssDNA at its 3′ extremity. The subject of the modelling in this example is the interaction of the RecA-DNA filament with the DNA polymerase DinB.

#### 3.2.1 Building-Block Scaffold

The modelling steps described here consist of the refinement and validation of a coarse-grained scaffold structure defined in a preliminary modelling study (Tashjian et al., 2019) of the RecA*-DinB complex, which itself was built on experimental evidence that the coupling between recombination and synthesis occurs via direct binding of the DNA polymerase DinB to the RecA* filament (RecA nucleoprotein filament with three bound DNA strands) (Henrikus et al., 2020) (see also Godoy et al., 2007). This structure was a coarse-grained model obtained using BioSpring, an interactive modelling facility also used in the RyR1 modelling presented above. The structure also integrated a structural building block RecA* derived from the model of early RecA-ssDNA-dsDNA intermediate created in 2015 (Yang et al., 2015). Indeed, the striking similarity between that RecA* model and the recently published Cryo-EM structure of short post-strand-exchange intermediates (Yang et al., 2020) provided further impetus for the current integrated modelling.

#### 3.2.2 Coarse-Grained to All-Atom

While the coarse-grained representation used in BioSpring presents a direct correspondence to all-atom representation, which allows easy recovery of an all-atom model, that model needed refinement. This was notably the case for the DinB building block, for which the spring network used in BioSpring had allowed distortions in the secondary structures during the tight assembly process.

We reintroduced individual secondary structure elements and loops taken from the DinB crystal structure as described in the *Methods* section; thereafter energy minimization was sufficient to regularize their geometries.

#### 3.2.3 Mechanical Coupling Within RecA*-DinB Complex

**FIGURE 6:**
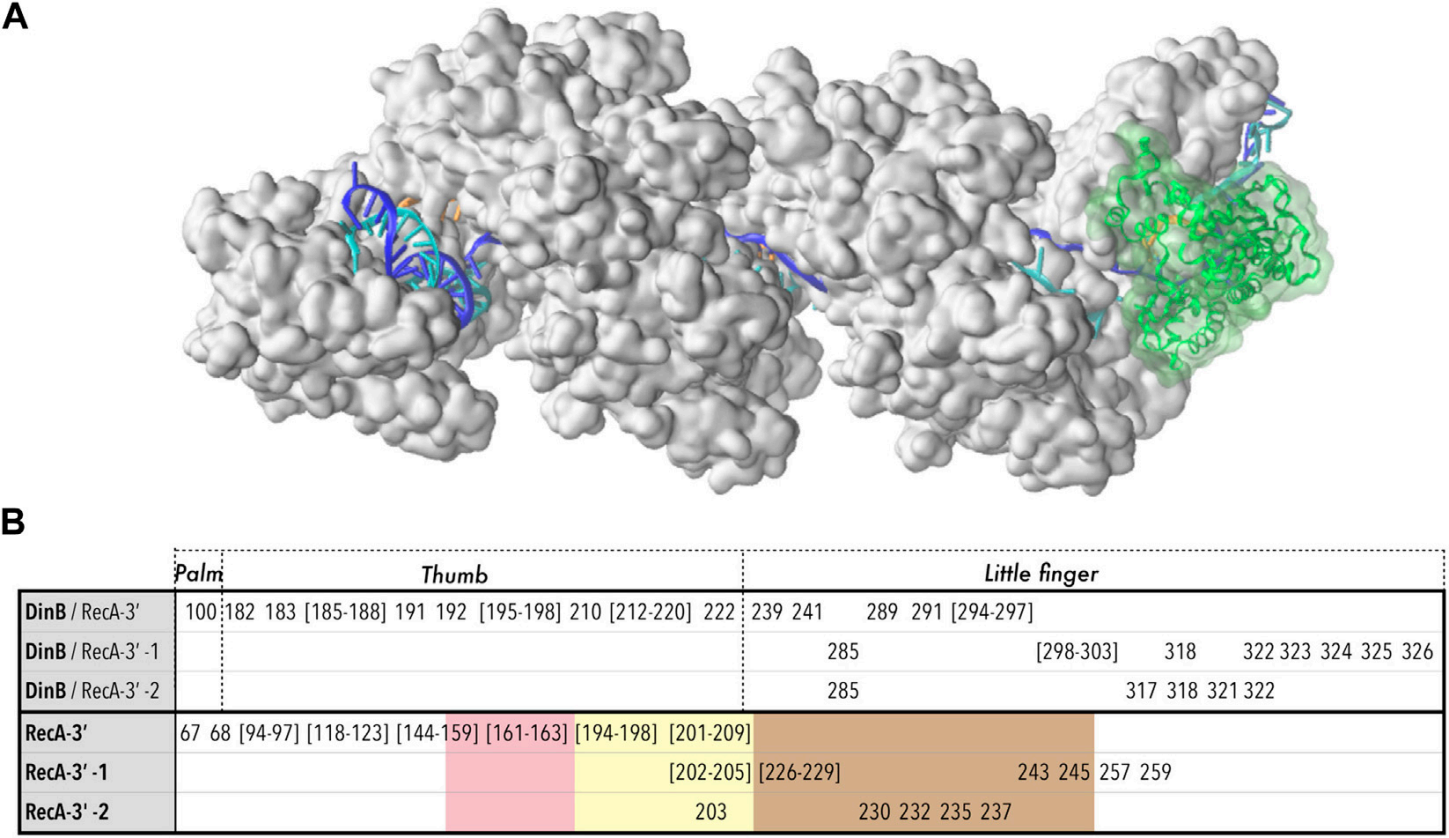
Model structure of the RecA*-DinB complex, after 270 ns molecular dynamics simulation. **(A)** The protein part of the RecA* filament (close to three filament turns) is formed by 17 RecA proteins shown in surface representation, in white. The filament binds three DNA strands (cartoon representation) organized as a D-loop: the ssDNA bound to the filament in site I, or primer (orange) binds the complementary or template strand (blue) in the region where that strand is incorporated in the filament, forming a stretched/unwound heteroduplex; beyond the initial dsDNA entry point in the filament in 5′ and its exit point in 3′, the complementary strand remains paired with its initial partner or leading strand (cyan) in a relaxed B-form. The junctions between the stretched and relaxed regions form kinks. The DinB protein is represented in cartoon and transparent surface, in green. **(B)** Residues involved in the interface between DinB and the last three RecA monomers starting from the 3′ extremity (RecA-3′, RecA-3′ -1, RecA-3′ -2). For DinB, correspondence with the “Palm”, “Thumb” and “Little finger” domains is reported; for RecA, the residues belonging to loops L1 (pink), L2 (yellow) or the LexA binding loop (marron), are indicated using color code shading. The same color code is used in Figure 5.

We subjected the all-atom starting model to 270 ns of molecular dynamics simulation in explicit solvent. Over the timecourse of the simulation, the filament flexes somewhat, with the overall end-to-end distance reducing by 10%, but no tendency for dissociation of the polymerase was observed (Figure 6A); the total number of DinB-RecA* interface contacts decreased by about 15% in the first 70 ns, then remained stable throughout the last 200 ns of the trajectory (Supplementary Figure S8). This indicates that the large majority of DinB/RecA contact regions from the preliminary modelled structure (Tashjian et al., 2019) were preserved in the highly crowded environment of the complex.

**FIGURE 5:**
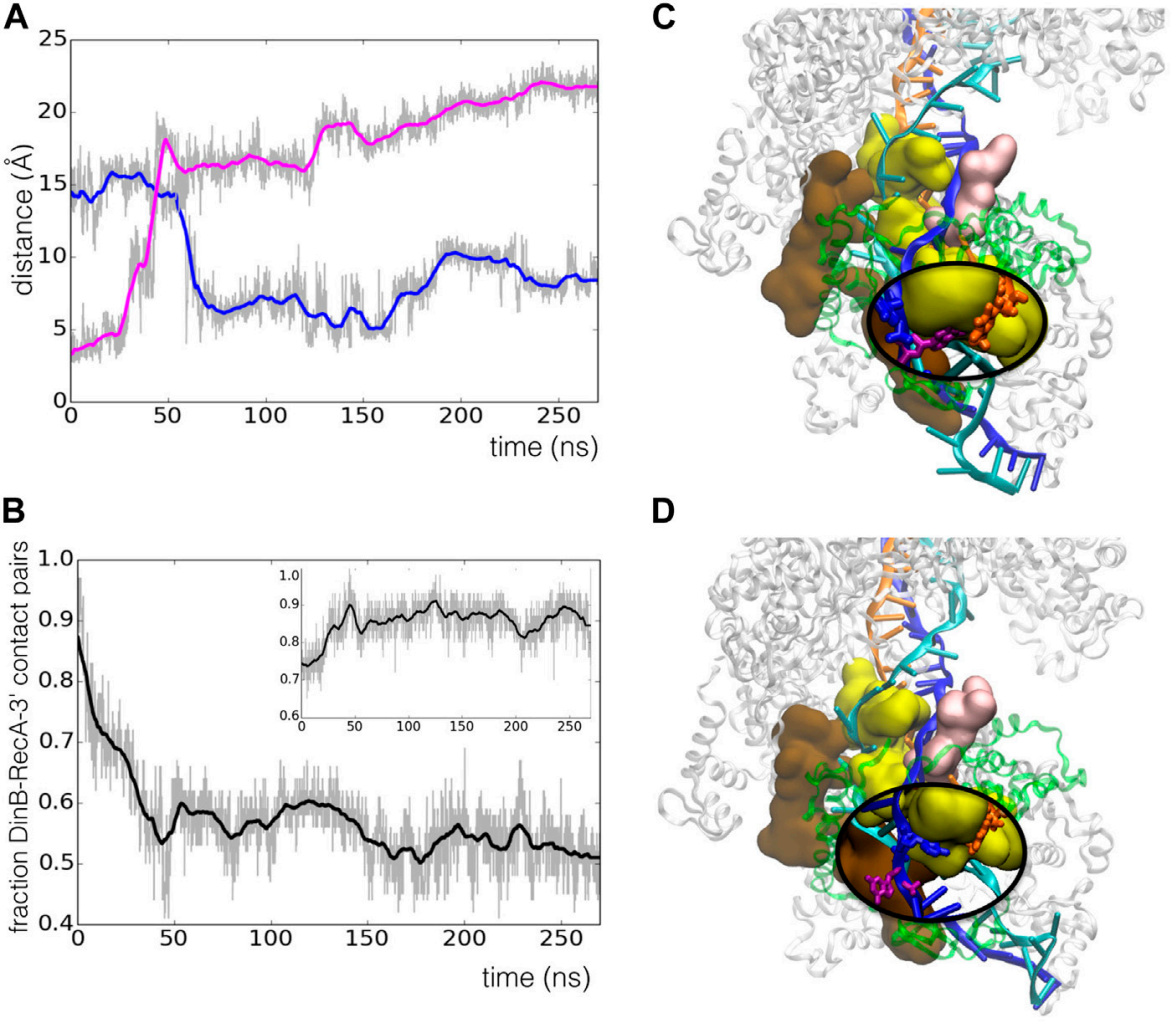
Spontaneous register shift to the correct base pairing in the RecA*-DinB complex during 270 ns MD simulation. **(A)** Time evolution of the distance between atom O4 of T_53_ in the primer strand and atom N1 of either A_12_ (blue line) or G_11_ (magenta line) in the template strand; $G_{11}^{\text{templ}}$ , initially close to $T_{53}^{\text{prim}}$ , rapidly separates from that thymine after from 30 ns, while $A_{12}^{\text{templ}}$ initiates a quick approximation to the thymine starting from 50ns simulation time; $T_{53}^{\text{prim}}$ : $A_{12}^{\text{templ}}$ is the last base pair between the primer and the template strands in 3′, while G_11_ is the first base of the template strand that will be used in the primer elongation process. **(B)** Time evolution of the fraction of residue-residue contacts between DinB and the last RecA monomer in the initial structure, that is conserved during the MD run; the inset shows the evolution with respect to the structure at 45 ns (vertical broken line) **(C,D)** Snapshots of the system taken at 20 ns **(C)** and 145 ns **(D)** simulation time. RecA proteins and DinB are, respectively, represented with white and green ribbons; motifs from the last three RecA proteins that are involved in the interface with DinB are shown in surface representation and colored using the codes given in Figure 6B; the DNA strands are represented in cartoon with color codes as described in Figure 6A. In both **(C)** and **(D)**, an oval insert with magnifying glass effect enables visualizing the bases referred to in **(A)**, represented in licorice with $T_{53}^{\text{prim}}$ in orange, $A_{12}^{\text{templ}}$ in blue and $G_{11}^{\text{templ}}$ in magenta; in order to permit visualizing the bases which are deeply buried in DinB catalytic cleft, DinB is not represented in the inserts.

However, we observed spontaneous relaxation that took place during the simulation trajectory through concerted rearrangements of RecA motifs and DNA strands. The starting model presented a mismatch in the last heteroduplex base pair in the 3′ end due to an artificial shift in the heteroduplex register resulting from the preliminary modelling process, which had shifted the last several bases of the template strand with respect to the primer, such that $G_{11}^{\text{templ}}$ interacted with $T_{53}^{\text{prim}}$ , the last 3′-base of the primer strand (Figure 5B). From the point of view of sequence, G_11_ is the first base of the template strand that should assist the synthesis of the first added nucleotide in the polymerase catalytic site to extend the primer sequence (Figure 5C). Figure 5A shows how the mismatched $T_{53}^{\text{prim}}$ : $G_{11}^{\text{templ}}$ heteroduplex base pair spontaneously separated after about 50 ns, rapidly followed by the formation of the correct $T_{53}^{\text{prim}}$ : $A_{12}^{\text{templ}}$ base pair (shown in Figure 5D). This pairing shift comes with a sliding of the DNA template strand backbone (in blue in Figures 5C,D along the L2 loop of the last RecA monomer (in yellow in Figures 5C,D). This loop is deeply inserted in the DinB catalytic cleft. Figure 5B shows that this sliding occurs simultaneously with a shift in the interface between DinB and the terminal RecA monomer, that also takes place at 50 ns. At the same time, the interface loses up to 50% of its initial residue pair contacts, stabilizing to a new network of residue-residue contacts that is 86% conserved on average during the last 225 ns of the simulation (Figure 5B, insert). Recent studies, both on RecA filaments and on heterodimers, showed that stable interfaces typically conserve 70–90% of their interaction contact network when simulated at 300 K, due to thermal movements (Boyer et al., 2019; Prévost and Sacquin-Mora, 2021).

#### 3.2.4 RecA*-DinB Interface

Figure 6B lists the residues that participate in the interface between DinB and the last three RecA monomers at the filament 3′ extremity. The list has been established from a MD structure at 120 ns simulation time, therefore after the reorganization took place. Two DinB domains mainly participate in the three DinB/RecA interfaces, namely the thumb and the little finger domains, with the palm domain showing only marginal contribution (for definitions of these domains see Figure 6). Decomposing the contributions of DinB domains and RecA motifs testifies to the intricate association of DinB within the filament interior. While the thumb domain only interacts with the last RecA monomer at the 3′ end, the little finger domain contributes to all three DinB-RecA interfaces. Three residues of the little finger domain, namely Arg85, Lys318 and Trp322, simultaneously interact with the two RecA monomers situated 5′ of the terminal RecA. On the RecA side, the interface between DinB and the terminal RecA encompasses most of the L1 and L2 loops, flexible loops that participate in the binding of the DNA strands in the HR filament. Although the L2 loop is deeply inserted in the DinB catalytic cleft, its presence remains compatible with that of the primer and template strands while allowing strand adjustments during the MD trajectory as described above. L2 loops of the two non-terminal RecA monomers also partly contribute to the interface with DinB, which also includes the so-called LexA-binding loop, a hairpin motif that contributes to forming the secondary DNA binding site in the filament.

The model we present here of a putative intermediate of association between the RecA nucleoprotein filament and the DinB DNA polymerase offers a link between the recombination and the DNA synthesis activities in HR. *In vitro* studies have previously demonstrated that DinB associates with isolated RecA proteins, and the DinB interface with RecA has been characterized (Godoy et al., 2007) (See also Supplementary Figure S9). Most interestingly, the regions (or patches) of the DinB surface that interact strongly with RecA were mainly observed within the catalytic cleft, in the little finger (patch *Pa1*: 287–298) and in the palm domains (patch *Pa2*: 145–160). Our model of DinB interacting with RecA*/DNA identified patch *Pa1* as a major contributor to the interface between DinB and the last RecA monomer (Figure 6). More exactly, *Pa1* interacts with the L2 loop of that monomer, which is deeply buried in the catalytic cleft. In addition, while patch *Pa2* does not directly contribute to the interface in our model, some of its residues such as Phe147 are found within 6 Åof that same L2 loop. Further approach of the *Pa2* patch to the L2 loop would appear to be sterically hindered by the neighboring RecA monomers. These observations suggest that DinB interacts with RecA in a similar way whether the RecA is part of the nucleoprotein filament or free in solution (Godoy et al., 2007).

## 4 Discussion

The integrative modelling pipeline we describe here, as summarized in Table 1, aims at achieving coherence, completeness, and (meta-)stability in the generated models.

Coherence was a constant concern for building the RyR1 models starting from Cryo-EM data generated in different conditions. It was ensured by separately applying the modelling pipeline to each of the input data sets, resulting in five complete all-atom reference models of functional substates ranging from open to closed conformations. In order to take into account the enormous size of assemblies such as the RyR1 receptor, our protocol focuses on providing a general approach with computationally efficient steps such as interactive modelling and coarse-grained refinement. The pipeline also provides the possibility to limit the computational cost (e.g., by fitting to cryo-EM sub-maps) when performance might be critical. It should be noted that our protocol differs from that of Heinz et al. (2018), whose focus was on quantifying ion permeation and identifying pathways through the channel core. That work used molecular dynamics simulations as the main tool to support the refinement process, which is computationally expensive. It focused on modelling the closed stated based on cryo-EM data from (Efremov et al., 2015; Yan et al., 2015; Zalk et al., 2015), while an open-state model restricted to the central and channel domains was generated from the closed-state model using MD guided by the cryo-EM data of Georges et al. (2016).

### 4.1 Missing Regions and Interactions

The RyR1 receptor also illustrates how this pipeline addresses the challenges of obtaining complete models from incomplete structural data. All available experimental density maps of RyR1 lack density for large regions of the sequence. This may indicate disorder or flexibility in these regions. However it is now well recognized that even intrinsically disordered, flexible protein regions may play important functional roles. In tackling this problem, we observed that fully automated protocols could not handle such situations. For example, the use of MDFF was not always sufficient for generating the missing regions. Human interventions were required to make decisions and optimize the models, using available experimental data and additional sequence analyses. This phase of the modelling also highlighted the important question of the role of the BL region in RyR1, which exhibits distinct patterns and a high degree of conservation but for which no structural data is available. We hypothesize that this region contributes to the interaction with partner molecules, phospholipids, or other RyR1 subunits. These steps in the pipeline provided evidence in RyR1 for an additional transmembrane region, consistent with suggestions in the literature (Georges et al., 2016; Van Petegem, 2017). The additional TM helix was stable in the MD simulation step and appeared to reinforce intra- and inter-subunit packing of the transmembrane region by providing additional bridging interactions between neighboring subunits as well as between subunits and the membrane. Our initial models for this region could be used in further studies as structural templates for building full-length RyR2 and even RyR3 models.

We emphasize the power that interactive manipulation and visualization offers to this part of the integrative modelling pipeline, in addition to traditional modelling and visualization tools, when confronting such complex problems. Interactive approaches have existed for some time, but have not typically been present (or acknowledged) in integrative modelling studies. In cases such as the RyR1 and the RecA homologous recombination systems described here, the overall modelling pipeline involves results from automatic procedures that are explicitly coupled to, interpreted, and filtered through human interaction. For example, both the RyR1 receptor and the RecA-DNA-DinB complex required assembling entangled regions in the modelled complex. The release of steric clashes in many such cases is a tedious task but one that can be handled efficiently using interactive simulations. It might be pointed out that, in modelling, human input is ubiquitous and nearly unavoidable; a common example is the choice of restraints to be applied in modelling, even in such simple cases as energy minimization. The underlying hypothesis is that the ability to manipulate parts of the model as it is being built– to incorporate feedback on the modelling and to make decisions when there are multiple options or ambiguities– will enable the development of better models. Here, we made use of our in-house tool BioSpring at several points in the pipeline. Such approaches have occasionally been documented in the literature (Molza et al., 2014; Croll, 2018), but currently remain marginal. Since integrative modelling often means seeking the best compromise from different and sometimes conflicting sources of information about a given target, the pipeline we have developed explicitly acknowledges that the human operator in the modelling loop may be relied upon to make informed decisions in accordance with the biological data and the physical laws at play in the chosen modelling approach, e.g., in molecular dynamics simulation, constrained minimization, etc. (Lanrezac et al., 2021).

### 4.2 Roles of Assessment and Exploration of Model Dynamics

Simulating the dynamic nature of structural intermediates in the modelling process also plays an important role at different steps of our pipeline, where it serves multiple but distinct purposes. Molecular dynamics simulation, whether obtained using all-atom or coarse-grained representations, allows checking the (meta)-stability of a generated model, of a folded protein for example, over a chosen timescale, as in the RyR1 receptor modelling. It also permits identifying dynamic evolution of models, which reflects plausible local dynamics and structural changes, as proved valuable in the RecA* system. However, even without costly exploratory MD simulations, normal modes analysis of model structures can provide insight into differences in their intrinsic vibrational dynamics depending on their functional state. For example, comparing the low-frequency vibrational modes for two different conformational states can provide information on large-amplitude, low-frequency mobility differences (e.g., Thomas et al., 1996; Batista et al., 2011). In the case of RyR1, we could examine such differences by exploiting the coherency obtained by modelling the different states of this receptor in order to establish comparisons. As shown, NMA also offers insight into the directions of conformational transitions between two conformational states, which reflects the power of looking to vibrational mode directions for understanding conformational change in biological macromolecules (Tama and Sanejouand, 2001) and predicting protein-ligand and protein-protein interactions (Moal and Bates, 2010).

Dynamic exploration by MD adds further value to integrative modelling through refinement of modelled structures, which can itself provide both better models and valuable insights into function. For example, when multiple building blocks, each refined independently of the others, are combined into a higher-order assembly, there is unavoidably a stress that develops at the interfaces. Such stress may be classified into two categories. The first is what might be termed a “residual” stress that must be resolved in order to obtain a stable interaction. Examples of this were seen in the steric clashes in the RyR receptor, and similarly in the interaction of the DinB crystal structure with RecA-DNA, when assembling subunits in which loops and other motifs in the interface regions had been modelled independently. In our work these stresses were resolved by interactive molecular dynamics approaches that induced little overall change in the geometry of the assembly itself. In contrast, the RecA system revealed a second category of stress that may be linked to the function of the RecA-DNA-DinB assembly (Lu et al., 2019). Evidence of this “mechanical stress” was seen in the resolution of the mismatch of the last basepair of the heteroduplex DNA bound to RecA through unbiased MD simulation, via a register shift in the DNA pairing which resulted from a collective movement that spontaneously reduced the mechanical stress on the stretched DNA strands in the complex. This stress has been shown to play a major role in the recombination process through single molecule experiments (Conover et al., 2011; Danilowicz et al., 2012, 2014). A precedent for the functional role of stress was also seen in our earlier modelling study of the RecA-DNA interaction at the initial stage of the HR process, in which dynamic exploration of an integrative model resulted in spontaneous strand exchange (Yang et al., 2015), essentially mimicking the very fast event that stands at the heart of the HR process. Such functional conversion of mechanical stress could not have been deduced from static models such as the Cryo-EM structures recently published in Yang et al. (2020). The MD results obtained in the present study similarly suggest that tension-induced collective rearrangements in the DinB-RecA* system may play a role during DNA synthesis in the last step of homologous recombination.

Our modelling pipeline is seen thus to place fluid boundaries between validation, refinement and dynamic exploration steps. Indeed, information obtained in the course of an integrative modelling workflow can be useful to orient further exploration of functional mechanisms related to the complex under study. This was the case for both systems presented here despite their very different characteristics. In the case of the RyR1 channel, validation of the ensemble of conformational substates through the NM analysis provides information about the dynamic relationship between these conformational states and evidence of transitions between them. This information is a step in understanding the nature of the transition pathways between the functional states. In the case of the HR complex, the observations that we made during refinement appear to provide important indications for further mechanistic exploration, while validating the model in terms of its capacity to demonstrate the transmission of stress in the DNA to other components of the assembly.

More generally, workflows such as the one presented here provide insights not only into the structure and sequence properties of studied complexes, but also into their dynamics and function, as is required for drug design. Models obtained in this way can be used, for example, to infer putative drug-binding pockets. But the dynamics of pocket accessibility affects its druggability, and thus dynamic information clearly adds insight, for example for the development of new molecules aimed at restoring the closure of the mutant RyR1 channel without altering its function.

## 5 Conclusion

The increasing flow of experimental structural data calls for a new class of integrative modelling workflows combining a broad range of tools. We have shown that integrative modelling of individual macromolecular building blocks, even for very large systems such as the RyR ion channel, is now possible. Ultimately, the results obtained from such approaches on the RyR family will pave the way to improve our understanding of allosteric long-range gating of channel opening and ligand binding effects, which are essential for drug development to treat RyR channelopathies. Further integrative modelling applications involve how such building blocks are assembled into higher-level organizations. Our case study of the complex homologous recognition system shows that such approaches, requiring tailored modelling tools, can go very far in the exploration of structure-function questions in a reliable fashion, as the building blocks are mechanically coupled. This procedure permits emitting testable hypotheses for challenging systems that are difficult to address experimentally.

## Data Availability

The raw data supporting the conclusions of this article will be made available by the authors, without undue reservation.
