# Supplementary material for: Building Biological Relevance Into Integrative Modelling of Macromolecular Assemblies
Source: Front Mol Biosci. 2022 Apr 11;9:826136. doi: 10.3389/fmolb.2022.826136 (PMC9035671; doi:10.3389/fmolb.2022.826136)
Supplement: Supplementary file 1 [file DataSheet1.pdf]

frontiers

Supplementary Material

1 INTEGRATIVE MODELLING OF RYR1

1.1 Figures

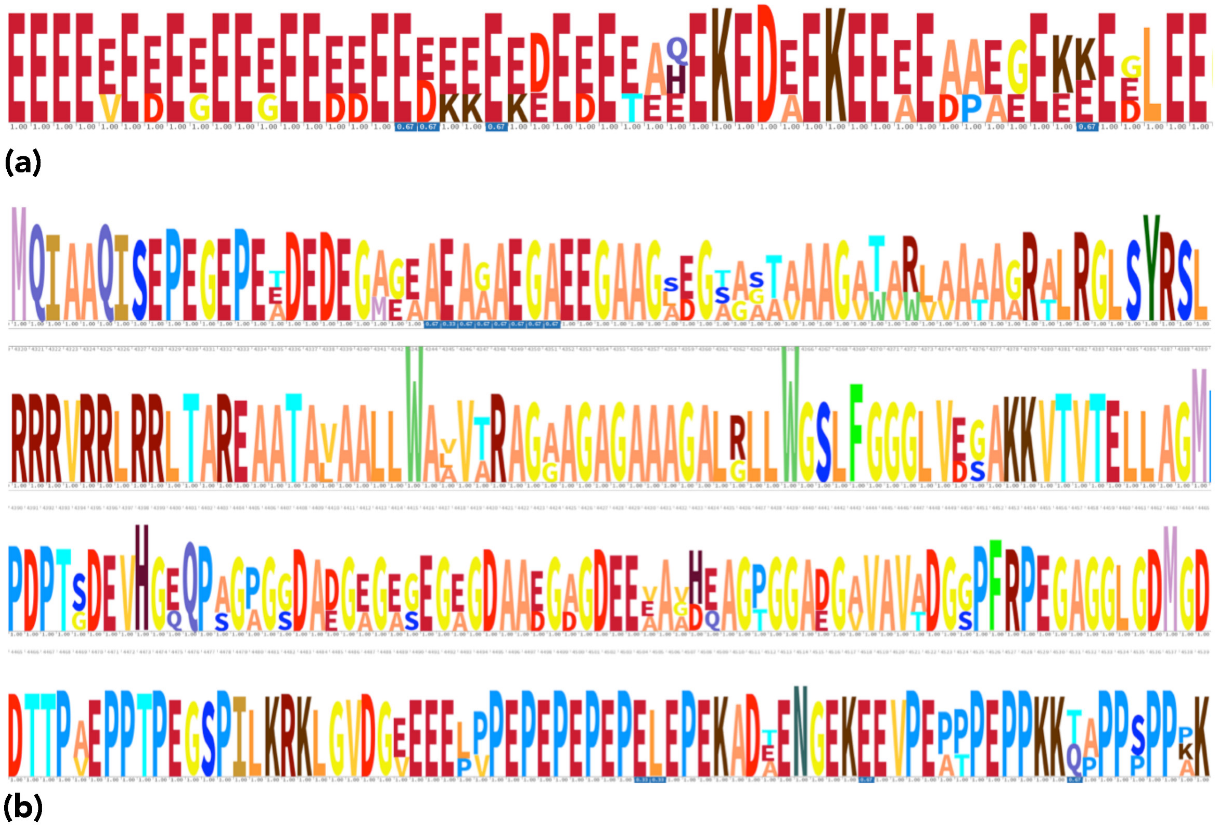

Figure S1. Sequence logo of selected regions of RyR1 homologs (a) Sequence logo of the glutamate-rich loop (residues 1875 to 1921) for human, rabbit, and mouse RyR1 homologs. (b) Sequence logo are displayed for the long unstructured region or big-loop (BL) connecting the cytoplasmic and transmembrane domains of human, rabbit, and mouse RyR1 homologs (residues 4254 to 4539). The height of the stack corresponds to conservation and the size of each residue letter is proportional to the frequency at this position.

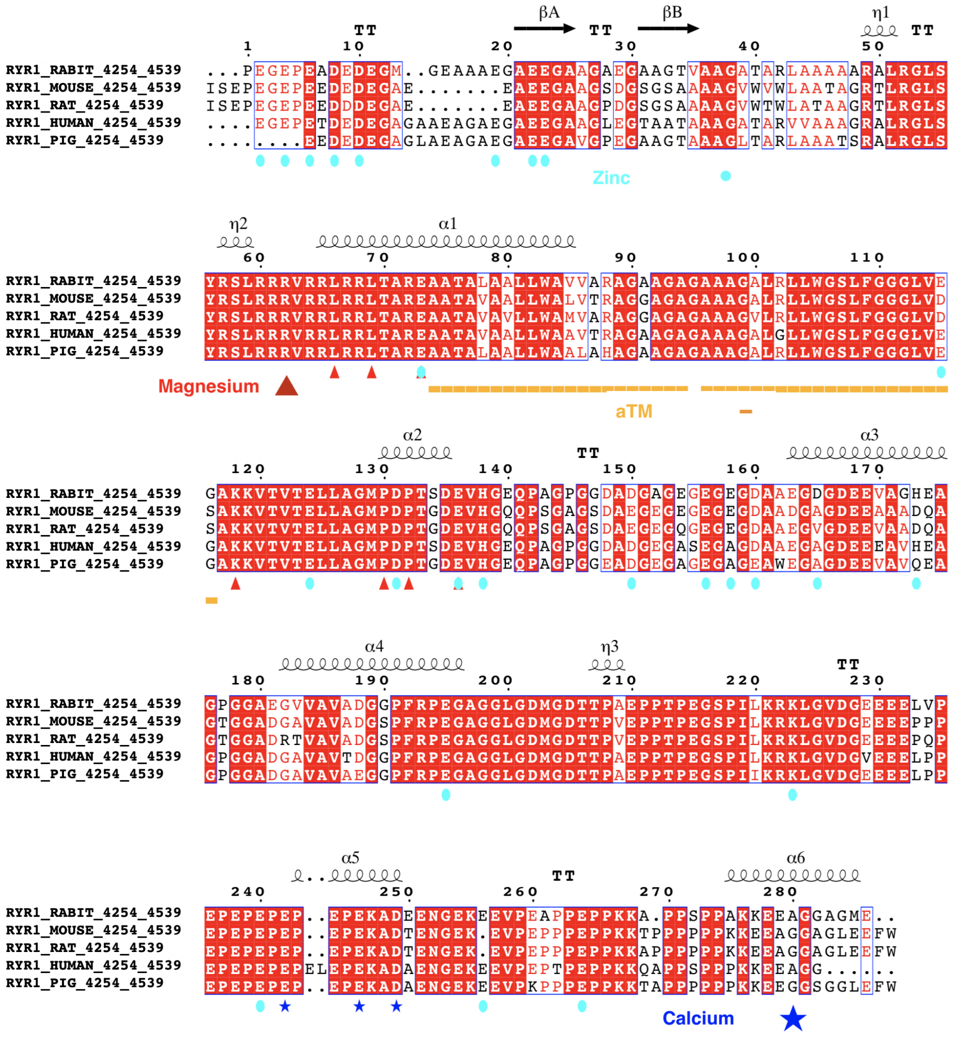

Figure S2. Multiple sequence alignment of the long region between residues 4254 and 4539, connecting the cytoplasmic and transmembrane domains for rabbit, mouse, rat, human, and pig RyR1. Note that some charged residues are conserved. The zinc, magnesium, and calcium binding sites are indicated. Secondary structure assignments are displayed, as is the predicted additional transmembrane helical portion.

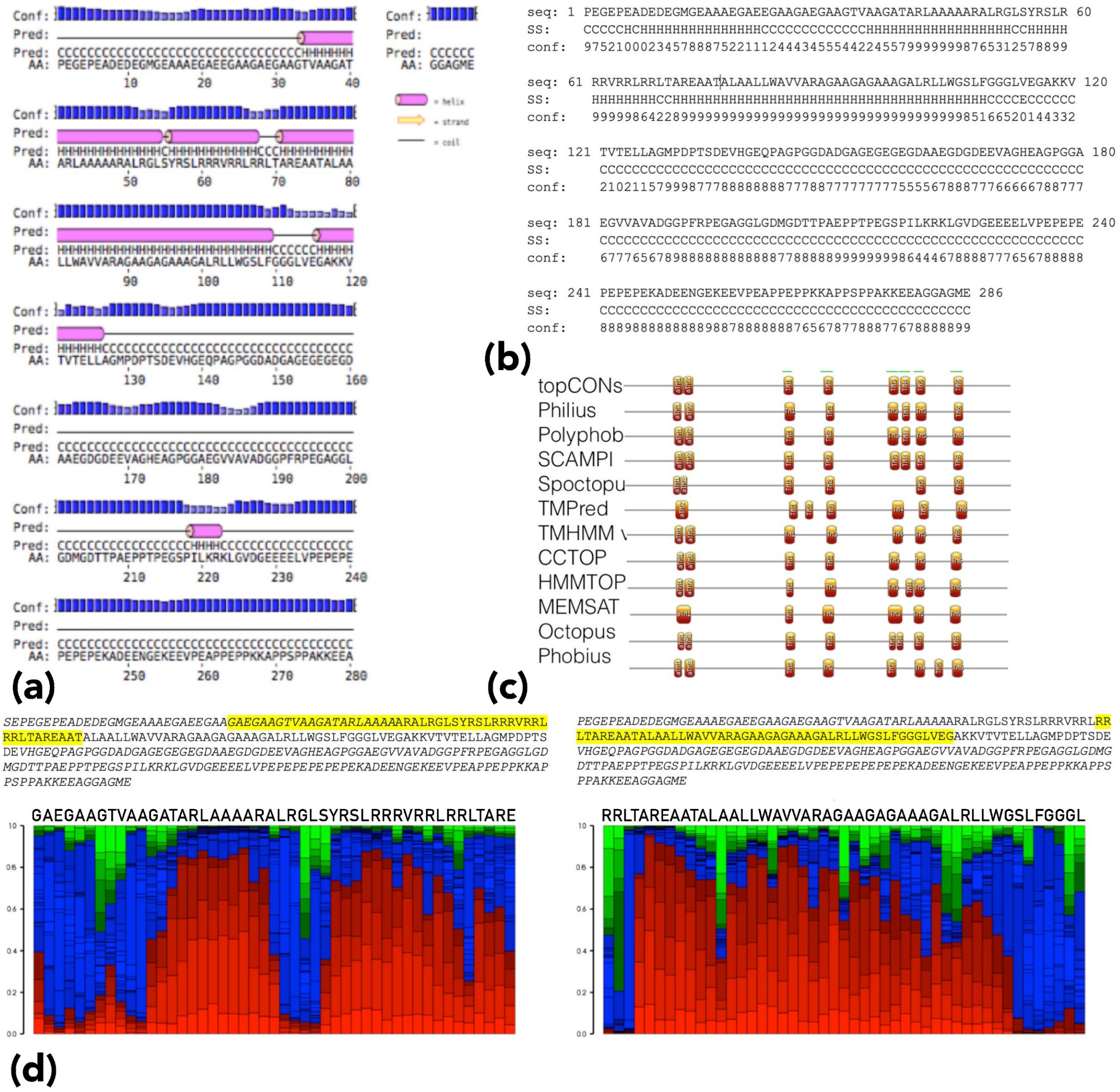

Figure S3. (a,b) PPSIPRED secondary structure predictions for the long disordered region between residues 4254 and 4539, connecting the cytoplasmic and transmembrane domains. In (a), 'Pred' is the predicted secondary structure type. 'AA' is the target amino acid sequence for the prediction; 'Conf' is the confidence of the prediction, either depicted graphically (a) or with numbers (b). (c) Comparison of transmembrane helix predictions from several servers for the long disordered region between residues 4254 and 4539, connecting the cytoplasmic and transmembrane domains. Green lines show the experimental mapping of transmembrane helices. All tools detect an additional helical region on the left side of the graph. (d) Results of PEP-FOLD modelling of two adjacent predicted transmembrane helical regions. The top row shows the underlying sequence segments. The graphical plots below represent the probabilities for each 'letter' in the Structural Alphabet (SA) at every position in the input sequence, with a sliding window of 4 residues per SA. In the profile, the red, green, and blue colors represent the helical, extended, and coiled regions, respectively.

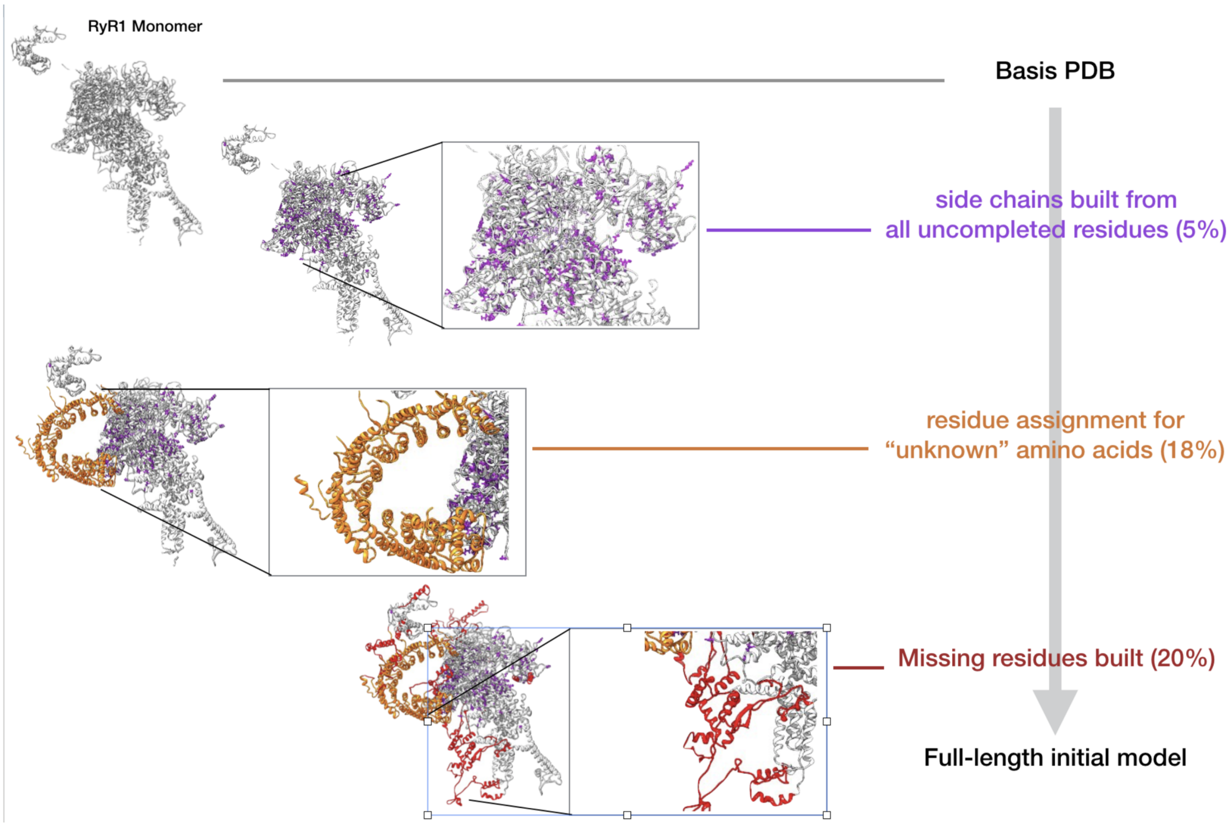

Figure S4. Overview of the steps required to model RyR1 in atomistic detail. For clarity, only one subunit is shown. Each step of the process for (re)building the residues is highlighted in color. Purple residues represent all missing side chains that have been built, orange regions indicate unknown residues that should be fully modelled, and red color indicates all remaining missing residues that have been built.

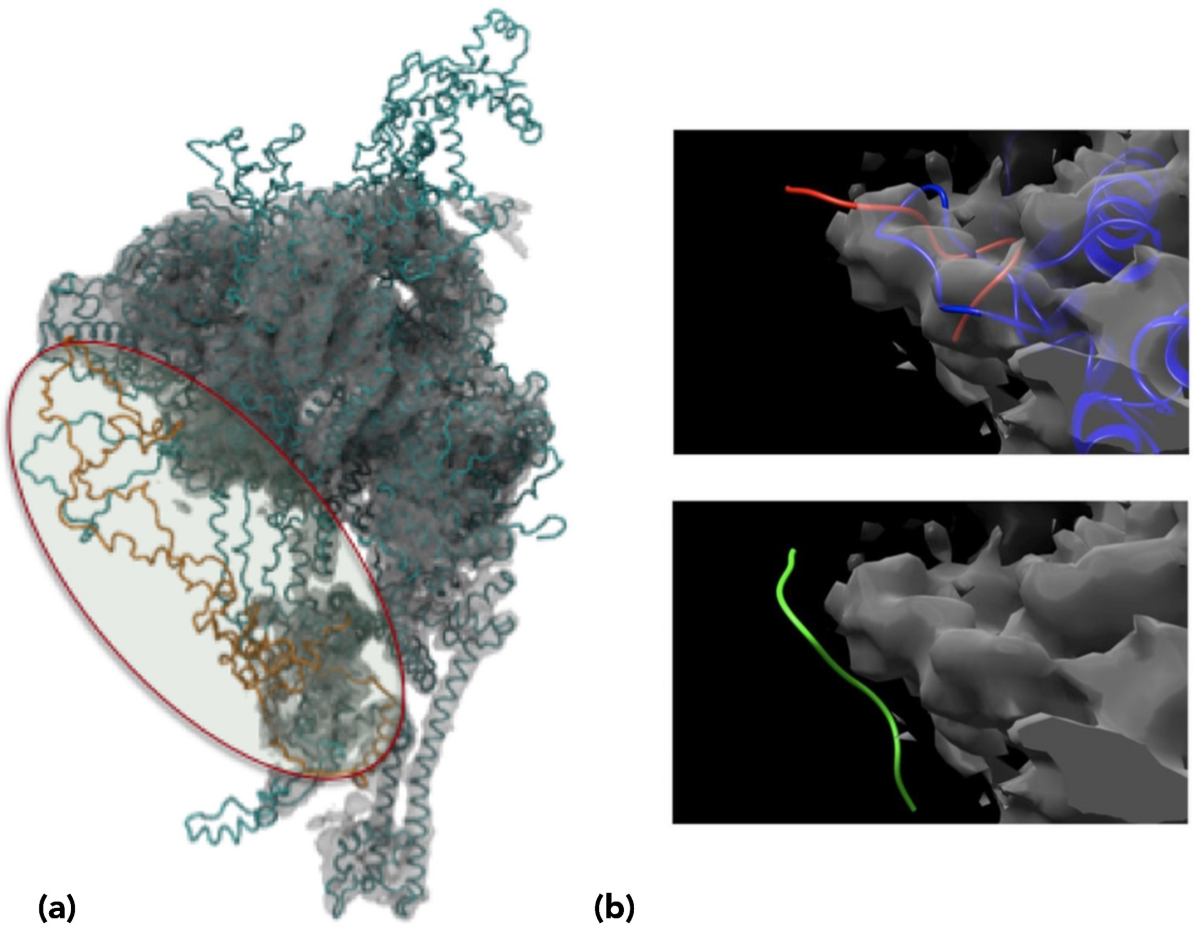

Figure S5. (a) Detailed view of one of the limitations of the MDFF method. Example of a long loop that did not fit in the map or was incorrectly positioned according to the MDFF method (red circle). For clarity, only one long loop is shown after the MDFF step. (b) Interactive and flexible loop optimization. Some residue coordinates were shifted after the MDFF step using the interactive and flexible fitting approach via BioSpring. Using this approach, we were able to shift the loops by adjusting them to the corresponding densities of the neighbouring subunits. The loop coordinates (residues 4290-4299) before (red, top panel) and after the BioSpring refinement step (green, bottom panel) are shown. For clarity, only the density map for one neighbouring subunit is shown.

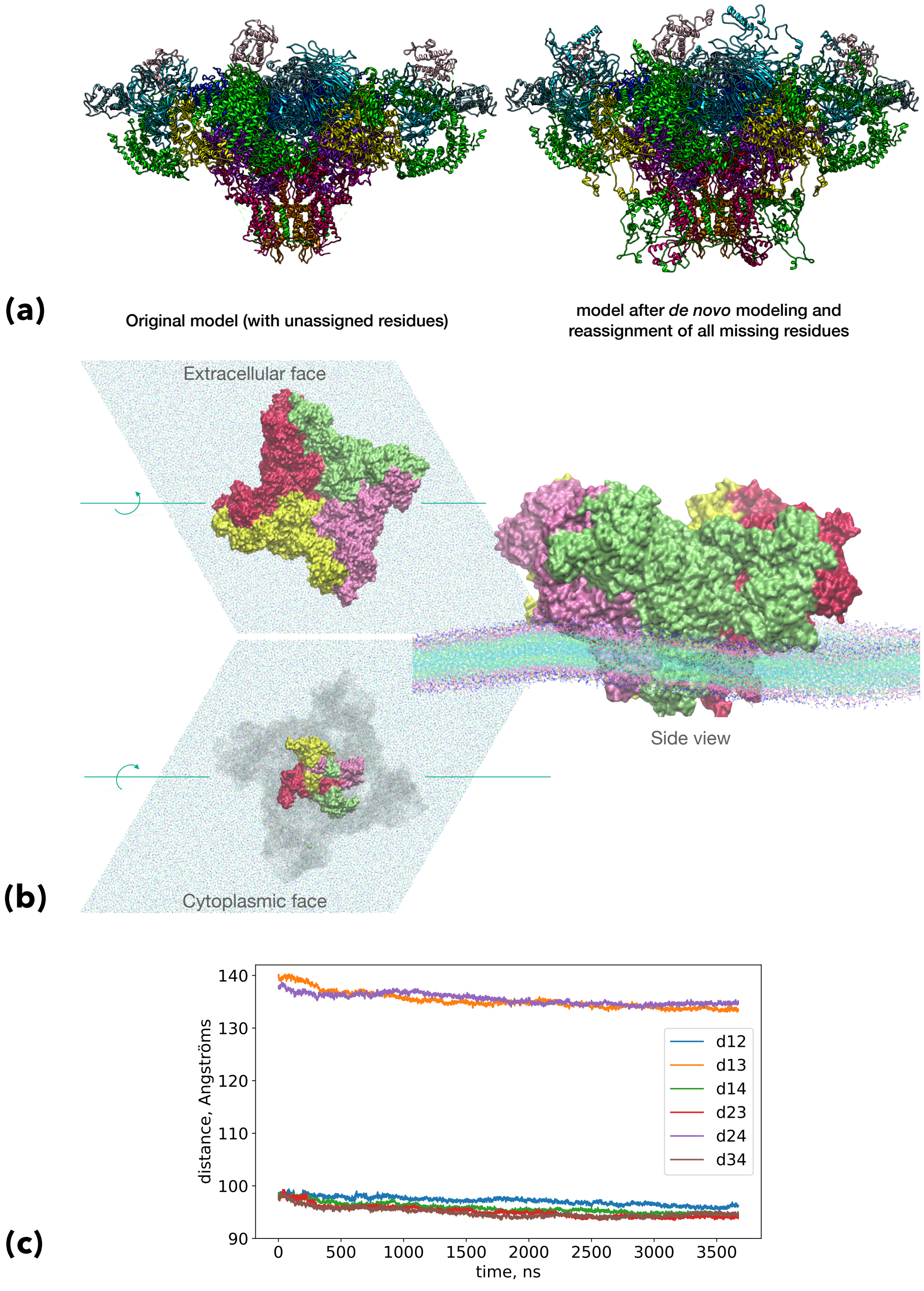

Figure S6. (a) Initial structure of the apo-RyR-1 receptor (left) and the final integrated model with all atoms (right). (b) Receptor colored by chain in its periodic, fully hydrated DOPC/DOPE membrane simulation cell. The view is from the two different faces of the membrane on the left and slightly zoomed in from inside the membrane on the right. (c) Center-of-mass subunit distances d_{ij} for all subunit pairs i, j as a function of time during 3.6 μs coarse-grained molecular dynamics.

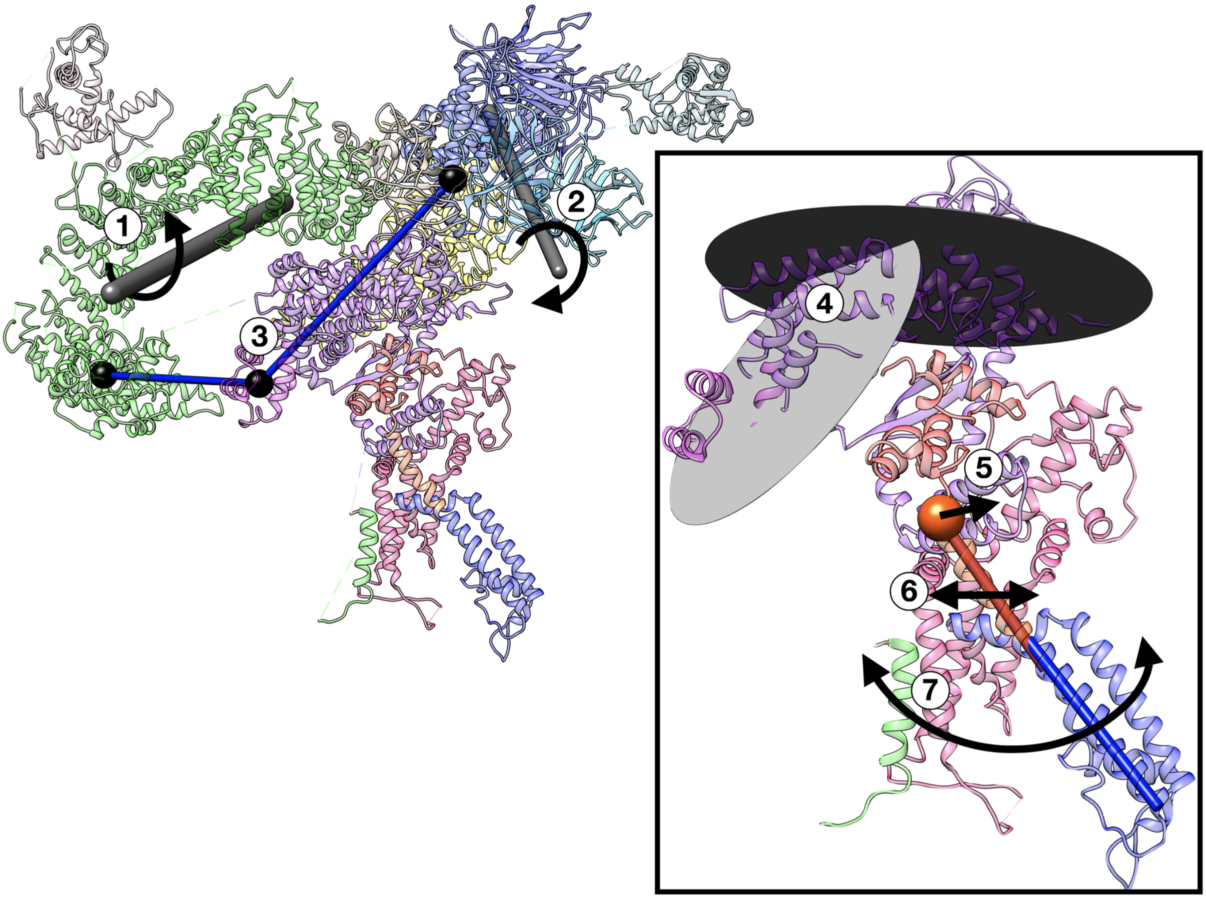

Figure S7. Motion criteria to filter out conformational changes involved in the RyR1 gating mechanism observed in protein dynamics studies using Chimera tools. For clarity, only one subunit is shown. To evaluate the relative motions of the transition pathways between the different conformations, we defined several movement types for the cytoplasmic part. *Blooming-shell motion* corresponds to the displacement of the atoms of the helical subdomains behind the pore. It uses 1) the rotation of the B-solenoid domain, 2) the rotation of the N-terminal domain, 3) the position of the C-terminal part of the N-terminal and the B-solenoid domains relative to the C-solenoid domain (black spheres with blue axes) and 4) the shifts of the N-terminal and/or C-terminal regions of the C-solenoid domain for the transmembrane part (inset), here called mini-blooming movements. For the blooming assessment of the channel models, we first define two planes using coordinates of the residue 3668-4178 by splitting the helical bundle containing the core solenoid and part of the EF-hand pair domains into two parts using Chimera tools. These planes are represented as disks with their centers aligned with the centroids of the residue regions 3668-3974 and 3974-4178, respectively. The crossing angle between the two planes was then measured. With the full-length models, blooming motions were defined as the displacement of atoms in three regions corresponding to the rotations of the N-terminal 1-506, bridging (bsol) 2386-3373 and core solenoid (csol) 3667-4115. *Breathing motion* refers to 5) the dilation in the putative membrane plane away from the central axis for each subunit, as measured by the relative movement of the cytoplasmic part of the S6 helix (S6c) to each other on different subunits. To assess this movement, we monitor the centers of mass of residue groups 4951-4956 in each subunit. *Tilting movement* refers to 6) the C-terminal part of S6 (orange axis) relative to the N-terminal part of S6 (blue axis). *Twisting movement* refers to 7) the rotational movement of the pore or TMD about the membrane normal axis. It is defined using the rotation of the ’pore region’ (S5-S6) about the z-axis. These motions may all contribute to some degree to channel gating with the twist mode being the most correlated to pore opening.

2 RECA*-DINB INTERACTIONS

2.1 Figures

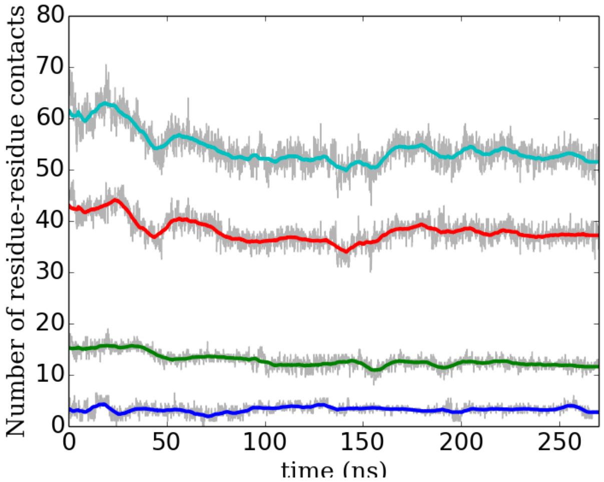

Figure S8. Time-evolution of the DinB-RecA interfaces. Number of residue-residue contacts between DinB and each of the three interacting RecA monomers in the filament, together with the total number of contacts, during the 270-ns of molecular dynamics trajectory. Two residues are considered in contact if at least two of their atoms are closer than 5 Å. After an initial decrease by about 15%, the total number of contacts remained stable during the last 200 ns.The curves relative to different interfaces are colored as follows: DinB-RecA-3’: red; DinB-RecA-3’-1: green; DinB-RecA3’-2: blue; total DinB-RecA: cyan.

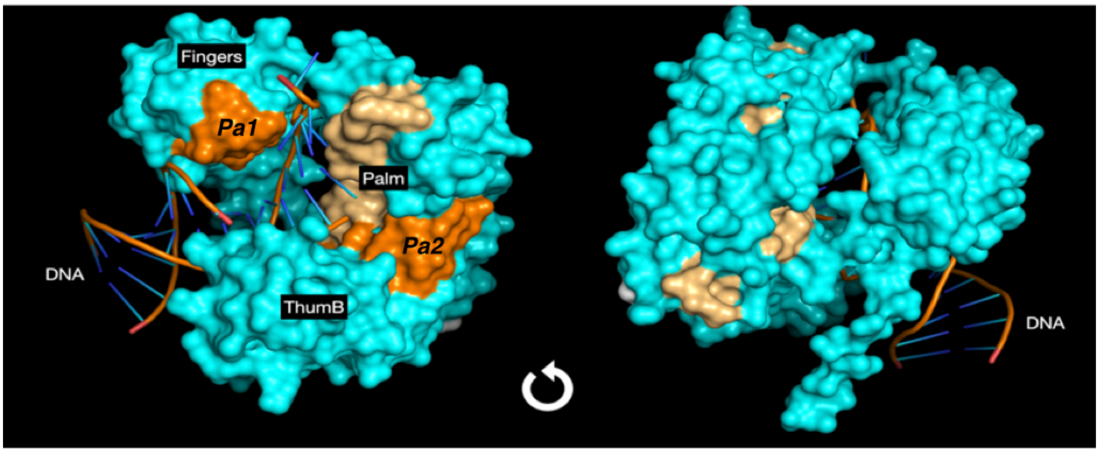

Figure S9. DinB interface with free RecA. Regions of DinB that have been shown to interact with free RecA proteins in a study by Godoy-Carter and collaborators (Godoy et al., 2007). Correspondence with the DinB interface regions in the DinB-RecA* model are discussed in the text. Dark brown colored areas shown putative strong interactions while light brown ones show weaker ones. The RecA interacting areas are on one face of the DinB. The main structural areas of DinB are labeled. Interacting areas were identified by synthesizing 12 aminoacid long overlapping peptides from the DinB sequence on a membrane; the membrane was then probed with RecA and developed with anti-RecA antibody (Godoy et al., 2007). The structure was rendered with PyMol.

REFERENCES

Godoy VG, Jarosz DF, Simon SM, Abyzov A, Ilyin V, Walker GC. UmuD and RecA directly modulate the mutagenic potential of the Y family DNA polymerase DinB. *Mol Cell* **28** (2007) 1058–1070. doi:10.1016/j.molcel.2007.10.025.
